# A LINE-1 insertion situated in the promoter of *IMPG2* is associated with autosomal recessive progressive retinal atrophy in Lhasa Apso dogs

**DOI:** 10.1186/s12863-020-00911-w

**Published:** 2020-09-07

**Authors:** Rebekkah J. Hitti-Malin, Louise M. Burmeister, Sally L. Ricketts, Thomas W. Lewis, Louise Pettitt, Mike Boursnell, Ellen C. Schofield, David Sargan, Cathryn S. Mellersh

**Affiliations:** 1 Kennel Club Genetics Centre, Animal Health Trust, Lanwades Park, Newmarket, Suffolk CB8 7UU UK; 2 Department of Veterinary Medicine, University of Cambridge, Cambridge, CB3 0ES UK; 3 The Kennel Club, London, W1J 8AB UK; 4 School of Veterinary Medicine and Science, The University of Nottingham, Sutton Bonington, Leicestershire, LE12 5RD UK

**Keywords:** Canine, Dog, Progressive retinal atrophy, PRA, Canine retinal degeneration, Inherited, Photoreceptor degeneration, *IMPG2*

## Abstract

**Background:**

Canine progressive retinal atrophies are a group of hereditary retinal degenerations in dogs characterised by depletion of photoreceptor cells in the retina, which ultimately leads to blindness. PRA in the Lhasa Apso (LA) dog has not previously been clinically characterised or described in the literature, but owners in the UK are advised to have their dog examined through the British Veterinary Association/ Kennel Club/ International Sheep Dog Society (BVA/KC/ISDS) eye scheme annually, and similar schemes that are in operation in other countries. After the exclusion of 25 previously reported canine retinal mutations in LA PRA-affected dogs, we sought to identify the genetic cause of PRA in this breed.

**Results:**

Analysis of whole-exome sequencing data of three PRA-affected LA and three LA without signs of PRA did not identify any exonic or splice site variants, suggesting the causal variant was non-exonic. We subsequently undertook a genome-wide association study (GWAS), which identified a 1.3 Mb disease-associated region on canine chromosome 33, followed by whole-genome sequencing analysis that revealed a long interspersed element-1 (LINE-1) insertion upstream of the *IMPG2* gene. *IMPG2* has previously been implicated in human retinal disease; however, until now no canine PRAs have been associated with this gene. The identification of this PRA-associated variant has enabled the development of a DNA test for this form of PRA in the breed, here termed PRA4 to distinguish it from other forms of PRA described in other breeds. This test has been used to determine the genotypes of over 900 LA dogs. A large cohort of genotyped dogs was used to estimate the allele frequency as between 0.07–0.1 in the UK LA population.

**Conclusions:**

Through the use of GWAS and subsequent sequencing of a PRA case, we have identified a LINE-1 insertion in the retinal candidate gene *IMPG2* that is associated with a form of PRA in the LA dog. Validation of this variant in 447 dogs of 123 breeds determined it was private to LA dogs. We envisage that, over time, the developed DNA test will offer breeders the opportunity to avoid producing dogs affected with this form of PRA.

## Background

Canine progressive retinal atrophies (PRAs) are a group of hereditary, heterogeneous diseases characterised by the degeneration of rod and cone photoreceptor cells in the retina. Clinical signs of PRA in dogs are very similar to those of retinitis pigmentosa (RP), the equivalent human inherited retinal degeneration which affects 1 in 4000 humans worldwide [1]. Despite the identification of 271 genes associated with inherited retinal diseases, including RP (RetNet, the Retinal Information Network [2]), many patients still lack a molecular diagnosis. Many of these genes are shared between human and canine inherited retinal degenerations, making the dog an excellent naturally occurring animal model for retinal disease [3]. Variability in the age of onset, aetiology and rate of disease progression is observed in both human and canine inherited retinal degenerations. In both species, retinal rod and cone photoreceptor cells are implicated in disease and degenerate over time. Photoreceptors are positioned within the outer and inner segment layers and the outer nuclear layer (ONL) of the retina. Depletion of photoreceptor cells results in thinning of the ONL. Rod-cone degenerations are characterised by the initial loss of rod photoreceptors, followed by a reduction in cone function. In cone-rod degenerations, cone function is severely affected initially, followed by rods. In both human and canine retinal degenerations, a moderate to complete loss of vision is inevitable [4]. Electroretinogram (ERG) assessment is not routinely performed in dogs, so distinguishing between a rod-cone or cone-rod degeneration is difficult, although when night blindness is the first clinical sign observed a rod-cone degeneration is considered the most likely diagnosis. The lack of ERG assessment in dogs means ophthalmoscopic examination is often the sole diagnostic procedure employed. Clinical signs observed by ophthalmoscopic examination include vascular attenuation of retinal blood vessels, retinal thinning leading to hyperreflectivity of the tapetum and, in later stages, atrophy of the optic disc [5]. PRA affects over 100 breeds of dog and is heterogeneous between and within breeds [6]. Thus far, mutations in 32 genes have been associated with canine PRAs [7–30].

The British Veterinary Association/ Kennel Club/ International Sheep Dog Society (BVA/KC/ISDS) eye scheme in the UK [31] and the European College of Veterinary Ophthalmologists (ECVO) Eye scheme [32] are clinical eye screening schemes available to dog breeders and owners in Europe to screen for hereditary eye diseases in dogs that are intended for breeding. The Lhasa Apso (LA) dog is currently listed the BVA/KC/ISDS Eye Scheme, meaning it is the considered opinion of veterinary ophthalmologists in the UK that PRA is diagnosed often enough in the breed to be of concern, and LA breeders are thus advised to have their dogs’ eyes examined annually by a BVA/KC/ISDS panellist. Currently there are no treatments generally available for PRA in dogs. Research studies using mice and dogs have shown the effectiveness of gene therapy as a treatment for some retinal degenerations [33–37], and cell-replacement therapy for human RP patients is being explored using the CRISPR/Cas9 system to correct genetic mutations in human cell lines [38], but these studies are in their relative infancy. The development of commercially available DNA tests for PRA-associated mutations therefore play an important role in controlling PRA, by enabling dog breeders to avoid breeding clinically affected dogs and to reduce the prevalence of PRA in breeds at risk. Clinical eye screening complements the use of DNA tests, where the former can identify novel/emerging eye conditions for which a genetic variant has not yet been discovered, and the latter enables dog owners and breeders to use a one-off genetic test to determine their dog’s genotype with respect to a specific mutation and make informed breeding choices, before signs of the disease are apparent. This is especially important for diseases whose clinical signs do not present until later in life, past the typical breeding age of the dog. DNA tests can also identify individuals that are heterozygous for a recessive disease-associated mutation, which a clinical eye examination cannot.

The form of PRA in the LA described in this study presents with an autosomal recessive mode of inheritance; however the exact age of onset can be difficult to determine when the disease is progressive and owners may remain unaware their dog is affected until visual impairment becomes severe. Cases have been reported, yet no genetic risk factor identified [22, 39]. The aim of this study was to explore this genetically distinct form of PRA in the LA dog, and identify the causal variant.

## Methods

### Study population

All dogs were examined by a veterinary ophthalmologist through a clinical referral process or via the BVA/KC/ISDS Eye Scheme in the UK, or the European equivalent(s). Dogs with a PRA diagnosis were defined as “cases”. Ophthalmoscopic examinations of these dogs showed bilateral retinal atrophy, detecting widespread tapetal hyperreflectivity, and retinal vascular attenuation. In some cases, secondary bilateral cataracts were also observed. An arbitrary age of ≥8 years old was chosen for LA dogs without signs of PRA to be used as “controls” based on the age of diagnosis and the difficulty in collecting samples from very old control dogs. An age of diagnosis was known for 19 of the 21 cases, with ages ranging from 1.75–11.96 years with a median age of 7.11 years (interquartile range 5.01–7.99).

### DNA extraction and quantitation

DNA was extracted from buccal mucosal swabs using the QIAamp DNA Blood Mini or Midi Kits (Qiagen, Manchester, UK). DNA concentration and purity were determined using the NanoDrop 1000 spectrophotometer (Thermo Fisher Scientific, Loughborough, UK) and/or the Qubit Fluorometer with the Qubit dsDNA broad range (BR) Assay Kit (Invitrogen, Loughborough, UK). DNA samples with concentrations < 10 nanograms per microliter (ng/μL) were concentrated using MultiScreen-PCR96 filter plates (Merck Millipore, Watford, UK) or Microcon − 30 kDa centrifugal filter units with ultracel-30 membrane (Merck Millipore, Watford, UK).

### Exclusion of known retinal mutations

#### Generating PCR amplicons

Genotypes of 25 previously published retinal mutations (Supplementary Table 1A; 1B), were determined using a combination of PCR-amplicon sequencing, amplified fragment length polymorphism (AFLP) analysis or PCR followed by agarose gel electrophoresis. All primers were designed using Primer3 (32, 33) and obtained from Integrated DNA Technologies (IDT, Leuven, Belgium). HotStarTaq Plus DNA polymerase (Qiagen, Manchester, UK) was used for standard reactions. PCR products used for AFLP analysis were analysed on an ABI 3130xl genetic analyzer (Applied Biosystems, Loughborough, UK) using Hi-Di formamide (Thermo Fisher Scientific, Loughborough, UK) and GeneScan 400HD ROX dye size standard (Thermo Fisher Scientific, Loughborough, UK). To generate amplicons for pooled amplicon sequencing, 18 primer pairs were pooled and a multiplex PCR was performed. Multiplex PCR and thermal cycling conditions are listed in Supplementary Tables 2 and 3.

#### Next-generation sequencing (NGS) of PCR amplicons for known PRA mutations

Purification was carried out after each thermal cycling reaction using AMPure XP beads (Beckman Coulter, High Wycombe, UK), according to the manufacturer’s instructions, and using a ratio of 1:1.75 for beads: DNA-containing solution. Adaptor ligation was performed followed by amplification to create sequencing libraries. Five μL of each sample library was pooled and quantified using a KAPA library quantification kit, according to the manufacturer’s instructions (Kapa Biosystems, Massachusetts, USA). The final library was diluted to 15 picomoles (pM) and loaded into a 150 base pair (bp) v3 kit cartridge (Illumina, Cambridge, UK) for single-ended sequencing on the MiSeq sequencing platform (Illumina, Cambridge, UK). FASTQ files were aligned to the canine genome assembly CanFam3.1 (Sep.2011. Broad CanFam3.1/canFam3, Dog release 89) [40] using BWA, producing BAM files. BAM files were visualized in the Integrative Genomics Viewer (IGV) [41, 42].

Large insertions and deletions or variants within repetitive regions, applicable for a total of seven mutations, were genotyped using PCR amplification, followed by either AFLP analysis or visualisation on an agarose gel using gel electrophoresis. Locus/gene information and primers for each mutation screened are listed in Supplementary Table 1B.

### Genome-wide association study (GWAS)

Genotyping was carried out using the Illumina CanineHD array (Illumina, San Diego, CA) comprising 173,662 single nucleotide polymorphisms (SNPs) (Neogen, Lansing, MI). Genome-wide association mapping was performed by allelic association analysis using PLINK after filtering SNPs with a call rate of less than 97% and minor allele frequency less than 5%; and individuals with a genotyping call rate of less than 90%. Multi-dimensional scaling (MDS) plots and quantile-quantile (Q-Q) plots were generated using PLINK to assess for the presence of population stratification. Probabilities generated from GWAS data were adjusted for multiple testing using the PLINK Max(T) permutation procedure, and for population stratification and sample relatedness using Efficient Mixed-Model Association eXpedited (EMMAX) [43].

### Whole-exome sequencing (WES)

We utilised a canine-specific exome capture bait design for whole-exome sequencing (WES) (manufactured by Nimblegen, Roche, CA, USA) [44]. The LA was part of a previous WES PRA study of six breeds (three PRA cases and three controls over the age of 8 years from each breed) (unpublished). DNA was extracted from buccal swabs using standard protocols and samples were randomised for library preparation and sequencing with respect to breed and case-control status. Subsequently, 1.1 μg of DNA from each of the 36 dogs was sheared (Covaris focused ultrasonicator) to an average size distribution of 180–220 bp and fragmentation was assessed using a Bioanalyser, at the High-Throughput Genomics Group, Wellcome Trust Centre for Human Genetics, University of Oxford, UK. In-house library preparations were made using a KAPA library prep kit (Kapa Biosystems, Massachusetts, USA) and SeqCap EZ library SR protocol and associated reagents (Nimblegen, Roche CA, USA). Briefly, samples were end-repaired, A-tailed and ligated with Illumina indexes (Illumina, Cambridge, UK). A clean-up at each stage was done using AMPure XP beads (Beckman Coulter, High Wycombe, UK). Following adapter ligation, the subsequent clean-up incorporated a size selection stage (post-ligation clean-up followed by Dual-SPRI size selection (250–450 bp)). The libraries were then amplified. After clean-up and quality-control (QC) assessment of pre-capture libraries, individual libraries for the 36 dogs were pooled into four pools of nine libraries. The four pooled libraries were hybridised with the exome capture baits for 64–72 h at 47 °C. Following hybridisation, libraries were washed and bound to capture beads, and subsequently amplified, quantified and purified. Exome enrichment was measured using quantitative PCR (qPCR) of four loci by comparing pre-capture pools with post-capture pools. The average-fold difference for all four assays was 171-fold. A final quantification of the four pooled libraries was done by qPCR. Paired-end sequencing (100 bp reads) was carried out on four lanes of an Illumina HiSeq2000 at the High-Throughput Genomics Group, Wellcome Trust Centre for Human Genetics, University of Oxford, UK. The average library read depth for the LA was 46X. Sequence reads were aligned to the canine reference genome (CanFam 3.1) using BWA [45] and SNP/insertion-deletion (indel) calls were made using GATK v3.6 [46, 47].

### Whole-genome sequencing (WGS) and variant filtering

Illumina sequencing of a TruSeq Nano library on a HiSeq X sequencing platform was conducted by Edinburgh Genomics, University of Edinburgh, UK, and generated a dataset of approximately 30X read depth. Reads were aligned to the canine reference genome (CanFam3.1) using BWA-MEM [45], variant calls were made using GATK v3.6 (HaplotypeCaller) and base quality score recalibration, indel realignment and duplicate removal performed [47]. SNP and indel discovery was performed using standard hard filtering parameters or variant quality score recalibration according to GATK Best Practices recommendations [46, 48]. Sequencing reads and variants were visualised manually in IGV [41, 42] across the defined disease-associated region from GWAS analysis and compared to 102 genomes from non-breed matched controls. Genomic Variant Call Format (VCF) files from 114 genomes were combined by HaplotypeCaller into a multi-sample VCF file. Cross-genome analysis was performed on the merged VCF file after annotating variants using Variant Effect Predictor (VEP) [49]. Variants from whole-genome sequencing (WGS) data were filtered appropriately for a recessive condition, i.e. homozygous in affected individuals only and allowing for control dogs to be heterozygous or homozygous for the alternate allele. An in-house analysis pipeline generated an effect-score for each variant, depending on its predicted severity/impact on protein sequence and whether it is deleterious. Scripts are publicly available in GitHub (https://github.com/AHT-CanineGenetics/Scripts/tree/hitti-malin_BMC). High-effect-score variants included those resulting in premature start/stop codons, splice site variants, nonsense and missense variants, frameshift variants, and in-frame deletions.

### Characterisation of the *IMPG2*-LINE-1 insertion

The length of the long interspersed element-1 (LINE-1) insertion was estimated by PCR using primers, forward 5′-CCAGGCCTCATGTTTAATAGC-3′; reverse 5′-GCACTGTTGGGTTCTTGGATA-3′, and conditions listed in Supplementary Tables 4 and 5. PCR products were amplified using PrimeSTAR® GXL DNA Polymerase (Takara Bio Europe, Saint-Germain-en-Laye, France) and separated using agarose gel electrophoresis. PCR products were also generated in the same way for next-generation sequencing (NGS) to determine the LINE-1 DNA sequence. Long PCR products were purified and prepared for NGS on a MiSeq platform using the methods previously described. De novo assembly was performed using SOAPdenovo [50].

### Variant screening

Candidate variants within the disease-associated region were genotyped in PRA cases and controls. Primer sequences are listed in Supplementary Table 6. Genotyping of the LINE-1 insertion in the interphotoreceptor matrix proteoglycan 2 (*IMPG2*) gene by AFLP was performed using PCR amplification using primers and assay details listed in Supplementary Tables 6, 7 and 8, followed by combining 1 μL of PCR product with 10 μL of a Hi-Di formamide (Thermo Fisher Scientific, Loughborough, UK) and GeneScan 400HD ROX dye size standard (Thermo Fisher Scientific, Loughborough, UK) mix to assess on an ABI 3130xl genetic analyzer (Applied Biosystems, Loughborough, UK). Probes for allelic discrimination assays were PrimeTime ZEN double-quenched qPCR probes containing a 5′ fluorophore, 3′ Iowa Black® FQ (IBFQ) quencher and proprietary, internal ZEN™ quencher. A 5′ HEX™ fluorophore was used to determine the reference allele and a FAM™ fluorophore to label the alternate allele (Supplementary Table 6). Individual PrimeTime assays were re-suspended in ultrapure water to a 10X mix and combined. Allelic discrimination assays were carried out using KAPA probe fast qPCR master mix (2X) (Sigma-Aldrich Company Ltd., Dorset, UK) on a StepOnePlus™ Real-Time PCR system (Thermo Fisher Scientific, Loughborough, UK) and results were analysed using ABI StepOne Software v2.3. PCR products to be used for Sanger sequencing were purified on a MultiScreen u96 filter plate (Merck Millipore, Watford, UK) and sequenced using the Sanger method using Bigdye v3.1 chemistry (Life Technologies Ltd., Loughborough, UK) and the following conditions: 96 °C for 30 s; 44 cycles at 92 °C for 4 s, 55 °C for 4 s, and 60 °C for 1 min 50 s. Isopropanol precipitation of sequencing reaction products removed excess reagents and precipitated DNA was resuspended in 10 μL Hi-Di Formamide (Applied Biosystems, Loughborough, UK). Sequencing products were separated on an ABI 3130xl genetic analyzer and data analysed using the Staden software package [51].

### In silico tools

The Ensembl genome browser (Dog release 89) [40] and UCSC genome browser [52] were used to obtain canine genome sequence (Sep.2011.Broad CanFam3.1/canFam3) to interrogate regions. Putative promoter regions were predicted using Gene2Promoter [53] and PromoterInspector [54]; and transcription factor binding sites (TFBS) using MatInspector [55]. Genotyping data were analysed using the PLINK software package [56]. Putative promoter regions were predicted using Gene2Promoter [53] and PromoterInspector [54]; and transcription factor binding sites (TFBS) using MatInspector [55]. NNSPLICEv.0.9 [57, 58] was used to evaluate splice site prediction to determine if intronic variants of interest caused disruption or introduction of exonic splicing or cryptic splicing.

### Breed relationships in a subset of PRA4-tested LA dogs versus a random set of KC registered LA dogs

To assess whether the Animal Health Trust (AHT) PRA4 DNA tested population was representative of the UK Kennel Club registered LA population, the pairwise kinship coefficients among a subset of PRA4 tested LA dogs born between 2009 and 2017 were compared to dogs randomly drawn from the Kennel Club registration database, also born between 2009 and 2017. Kinship coefficients between each of the dogs within each sample were computed [59, 60], and sample mean and standard deviation were calculated. Additionally, MDS plots were generated to depict relatedness within and between the AHT sample set and 1000 dogs randomly sampled from those born between years 2009–2017.

## Results

### Genome-wide association study (GWAS)

**Fig. 1:**
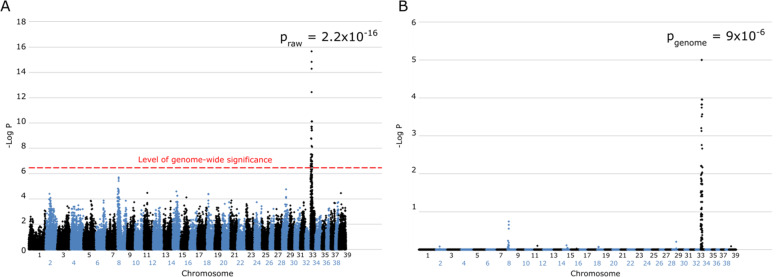
Manhattan plots of LA PRA GWAS. The level of genome-wide significance, determined by Bonferroni correction, is shown in (**a**) by a red dashed line. The signal remained significant after correcting for multiple testing by permutation (**b**)

A GWAS was conducted using 17 PRA cases and 27 controls. After QC filtering, 108,263 SNPs were included for the analysis of 42 dogs (15 cases and 27 controls). Analysis of GWAS data revealed a genome-wide significant association on canine chromosome 33 (CANFA33; −Log_10_ p_raw_ = 2.2 × 10^− 16^) (Fig. 1a). The signal remained significant after correcting for multiple testing (p_genome_ = 9 × 10^− 6^) (Fig. 1b). The MDS plot showed a similar distribution of cases and controls (Supplementary Figure 1). After correcting for population stratification and sample relatedness, the signal on CANFA33 remained statistically associated (*P* = 1.6 × 10^− 17^). Q-Q plots suggested potential population stratification with a moderately increased genomic inflation factor (λ = 1.36) which decreased to baseline (λ = 1.02) following corrections (Supplementary Figure 2).

**Fig. 2:**
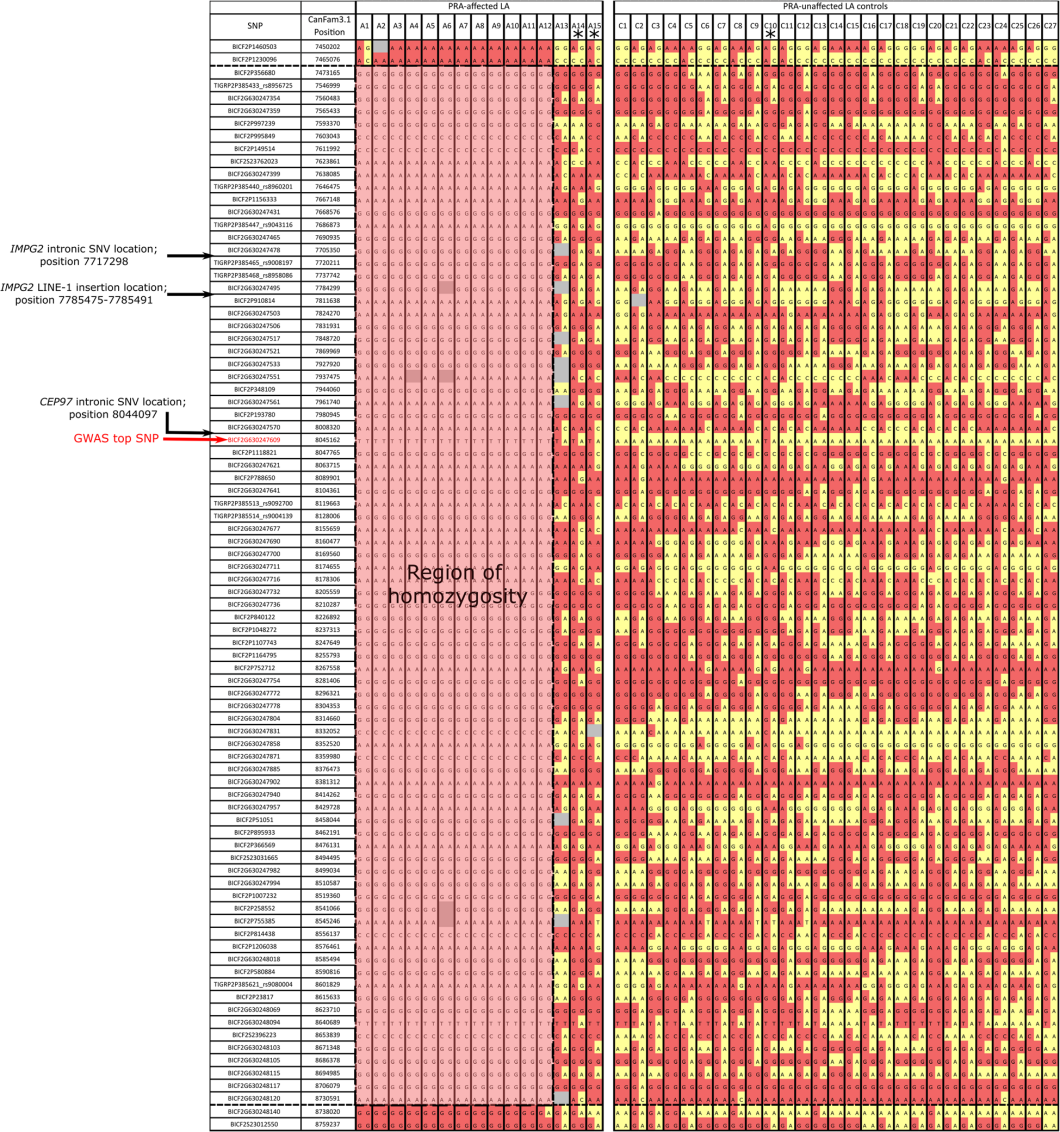
Homozygosity mapping of SNP markers to define the critical region. SNP markers surrounding the most associated SNP from the GWAS (SNP BICF2G630247609; text highlighted in red) in LA PRA cases (PRA-affected LA) (A1–15) and controls (PRA-unaffected LA controls) (C1–27) are shown. The yellow coloured boxes represent the reference alleles, the pink coloured boxes highlight the alternate alleles and grey boxes represent missing data. The 1.3 Mb critical region was homozygous in 12 of the 15 cases used in GWAS analysis, as shown by the shaded region between positions CANFA33: 7,465,076- 8,738,020. One LA control (C10) is heterozygous for this region, in addition to two of the affected LA that are not homozygous for the critical region, as marked by asterisks (*). Locations of the four variants later followed up after subsequent WGS analysis (LINE-1 insertion, the top associated GWAS SNP (BICF2G630247609), and intronic SNVs in *IMPG2* and *CEP97*) are indicated to show where these variants lie within the critical region

Visualisation of SNPs either side of the most associated SNP (SNP BICF2G630247609; *p*-value = 2.2 × 10^− 16^) in affected dogs sharing the disease-associated haplotype identified a disease-associated interval 1.3 megabases (Mb) in size that was homozygous in 12 of the 15 cases (Fig. 2).

The defined critical region harbours 21 genes, of which 12 are protein coding (Table 1). Two of these genes are potential candidates: interphotoreceptor matrix proteoglycan 2 (*IMPG2*) and centrosomal protein 97 (*CEP97*). *IMPG2* has previously been associated with autosomal recessive RP and vitelliform macular dystrophy (VMD) in humans [61, 62] and is therefore a strong candidate gene for canine PRA. *CEP97* plays a role in centrosome function and ciliary formation [63] and although *CEP97* has not directly been implicated with human retinal degenerations, mutations in other centrosomal protein coding genes have been associated with both syndromic and non-syndromic retinal degenerations (*CEP19*, *CEP78*, *CEP164*, *CEP250* and *CEP290*) [64–75].

**Table 1:** Protein coding genes situated within the 1.3 Mb critical region. An asterisk (*) highlights genes previously associated with, or within a gene family associated with retinal degeneration in humans

Gene Name	Abbreviation
ABI family member 3 binding protein	*AB13BP*
Interphotoreceptor matrix proteoglycan 2	IMPG2*
SUMO1/sentrin specific peptidase 7	*SENP7*
tRNA methyltransferase 10C, mitochondrial RNase P subunit	*TRMT10C*
PEST proteolytic signal containing nuclear protein	*PCNP*
Zinc finger and BTB domain containing 11	*ZBTB11*
Centrosomal protein 97	CEP97*
Neurexophilin and PC-esterase domain family member 3	*NXPE3*
NFKB inhibitor zeta	*NFKBIZ*
Zona pellucida like domain containing 1	*ZPLD1*
ENSCAFG00000009584	N/A; no human orthologue
Ribosomal protein L24	*RPL24*

### Identification of candidate causal variants underlying the GWAS signal

**Fig. 3:**
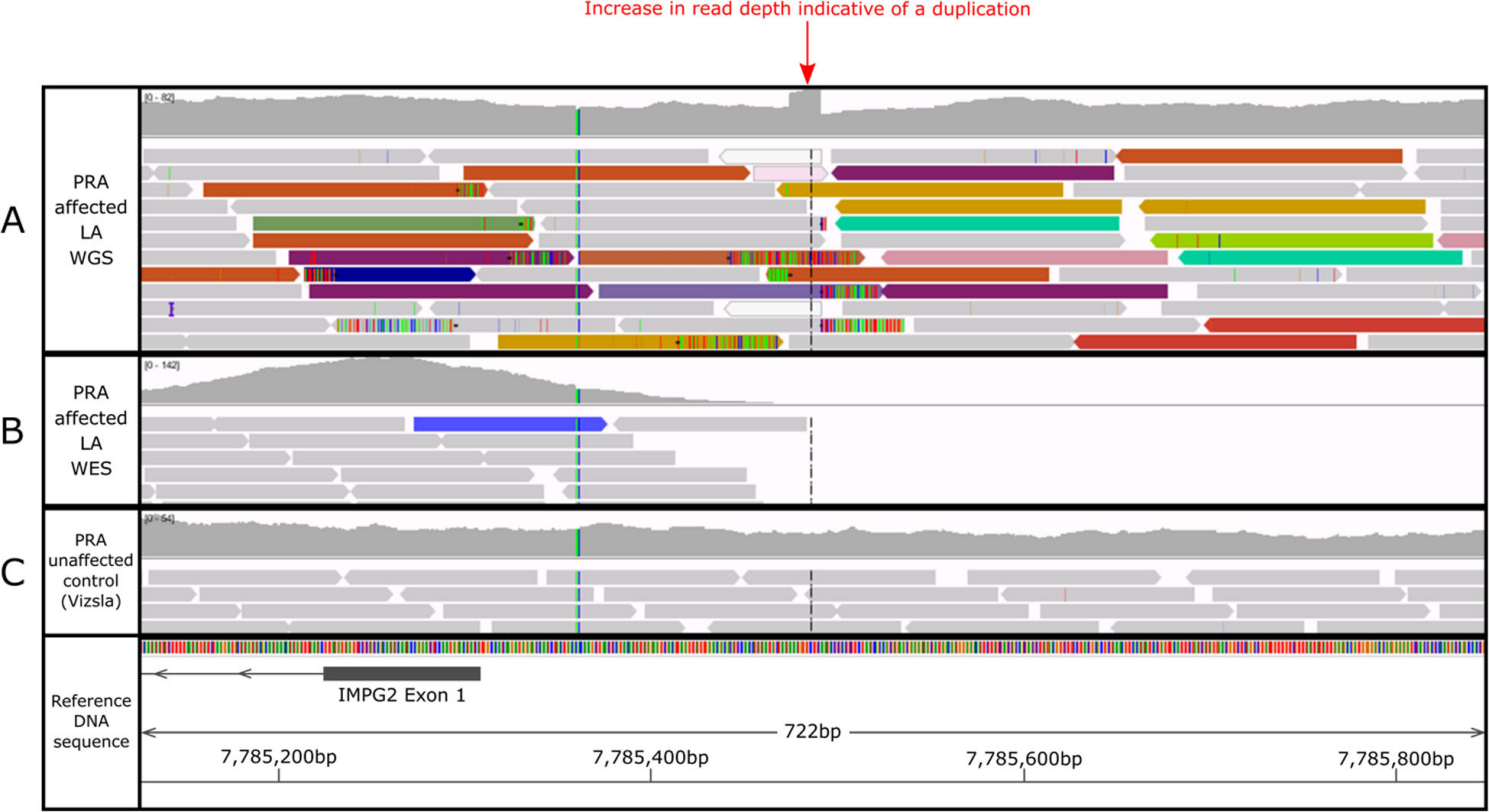
WGS (track **a**) and WES (track **b**) alignments over a 722 bp region on CANFA33 from a PRA-affected LA compared to control WGS data from a PRA-unaffected Hungarian Vizsla dog (example, track **c**). The identified LINE-1 insertion is upstream of the retinal candidate gene *IMPG2*. Grey sequencing reads show those aligning normally to the CanFam3.1 canine reference genome at this position. Coloured reads indicate that one of the paired sequencing reads aligns to this region on CANFA33, and the mate in this pair of sequencing reads aligns to another chromosome (each colour represents alignment to a different chromosome), consistent with the presence of a repetitive element insertion

From examination of WES data for three PRA-affected and three unaffected LA dogs, no exonic or splice site variants that segregated with PRA could be identified. The 1.3 Mb homozygous interval was therefore manually interrogated in WGS data of a PRA case using IGV software. A LINE-1 insertion was identified within the critical region in this PRA-affected LA, situated within 200 bp upstream of the interphotoreceptor matrix proteoglycan 2 (*IMPG2)* gene within the following coordinates: CANFA33: 7,785,475-7,785,491 (Fig. 3, track a). This insertion was not visible in the WES data of a PRA case (Fig. 3, track b). In control genomes, the insertion was not present. Variant filtering of WGS data identified two intronic single nucleotide variants (SNVs) situated in retinal candidate genes within the critical region: one in *IMPG2* (G/T SNV; CANFA33: 7717298) and one in *CEP97* (A/G SNV; CANFA33: 8044097*)*. In silico analysis concluded that neither the *IMPG2* or *CEP97* intronic SNVs are located within predicted donor or acceptor splice sites or nearby any splice site predictions. The locations of these two intronic SNVs within the defined homozygous critical region are highlighted in Fig. 2. The LINE-1 insertion was absent in WGS data from 102 individuals of 52 other breeds and 2 crossbreeds; WGS data from a Hungarian Vizsla dog is shown in Fig. 3, track c. Both intronic variants were looked for in the same 102 canine genomes. The *IMPG2* intronic SNV was absent in all 102 individuals and the *CEP97* intronic SNV absent in 101 individuals, with one Welsh Springer Spaniel dog identified as heterozygous for the SNV.

### Sequencing the LINE-1 insertion confirms a partial transposable element

**Fig. 4:**
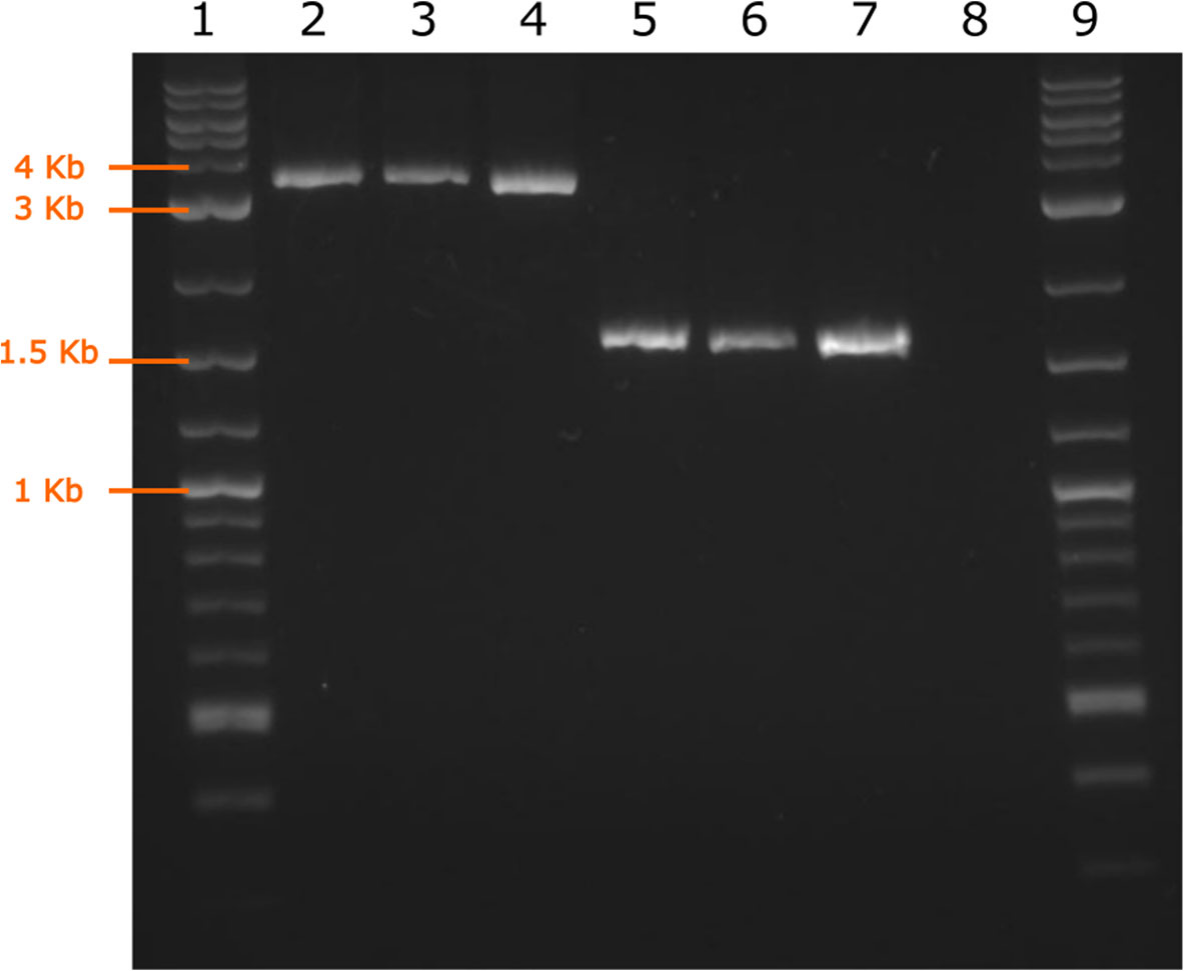
Agarose gel electrophoresis of three LA PRA cases (lanes 2–4), three LA PRA-unaffected controls (lanes 5–7), a negative control (lane 8) and a DNA marker ladder (lanes 1 and 9) (NEB). The expected PCR product size in unaffected controls is 1606 bp. The product containing the LINE-1 insertion is ~ 3.5 Kb on the gel, suggesting the LINE-1 insertion is ~ 1.9 Kb in size

Amplification by PCR across the LINE-1 insertion in three LA PRA cases and three LA controls suggested a size of 1.5–2 Kb (Fig. 4). NGS of the LINE-1 region confirmed an insertion of at least 1600 bp. The exact length of the poly-A tail could not be determined due to the low complexity of these short sequencing reads generated from the Illumina sequencing.

### Variant screening

To assess the concordance of the LINE-1 insertion with PRA, an AFLP assay was used to genotype 447 dogs of 122 breeds (Supplementary Table 9). Of the individuals in the GWAS dataset that passed QC, all 12 LA dogs that were homozygous for the defined critical region were clinically affected and homozygous for the LINE-1 insertion and in the control set, one heterozygote was present and the LINE-1 insertion was absent in the other 26 dogs of the control set. The cohort of additional controls included PRA cases of breeds related to the LA: five Shih Tzu dogs, seven Tibetan Spaniels and two Tibetan Terriers. All of these dogs were homozygous for the wild type allele.

Four out of the seventeen PRA-affected individuals included in the original GWAS dataset pre-QC filtering were not homozygous for the 1.3 Mb defined critical region. Presuming a single-gene disorder model, these four individuals were surmised to be suffering from a genetically different PRA and were therefore excluded from further analysis. Five additional PRA cases that were not included in the GWAS dataset were available to genotype for the LINE-1 insertion, the top associated SNP from the GWAS (BICF2G630247609) and both intronic SNVs in *IMPG2* and *CEP97*. In total, 59 LA dogs comprising 18 PRA cases and 41 controls were genotyped for these four variants in an attempt to assess which variant showed the strongest segregation with PRA (Table 2). One of the additional PRA cases (individual A18) was homozygous for the wild type allele across all four variants. Supplementary Figure 3 shows a schematic diagram of these four variant genotypes across the 59 LA dogs.

**Table 2:** Genotype distributions in LA PRA cases and controls for the four variants of interest

Variant	PRA-affected LA			PRA-unaffected LA			
	Alt/Alt	Alt/Ref	Ref/Ref	Alt/Alt	Alt/Ref	Ref/Ref	
*IMPG2* intronic SNV (chr33:7717298)	17	0	1	0	6	35	
*IMPG2* LINE-1 ins	17	0	1	0	6	35	
*CEP97* intronic SNV (chr33:8044097)	17	0	1	0	8	33	
BICF2G630247609	17	0	1	0	8	33	

### Promoter and transcription factor binding site predictions

**Fig. 5:**
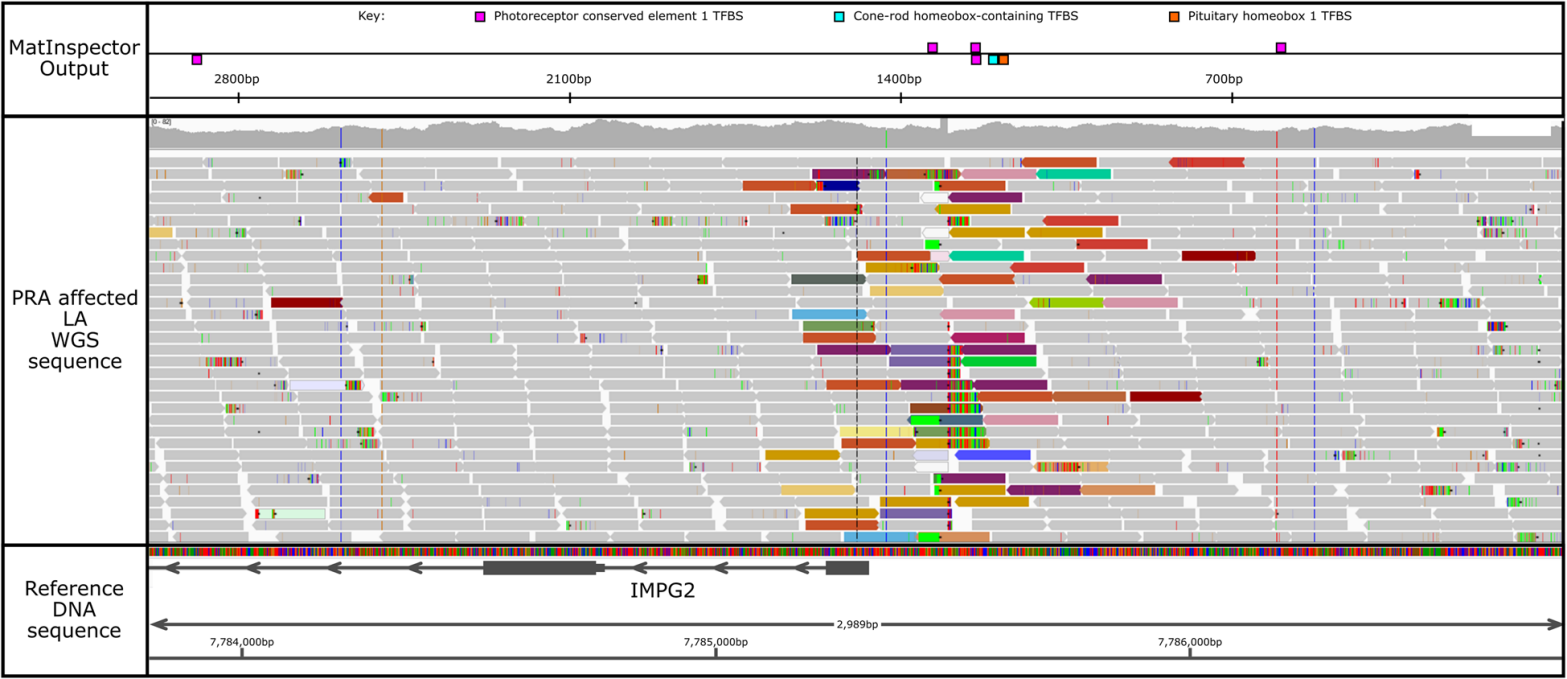
TFBS within the ‘V$BCDF’ matrix family from MatInspector within a 2989 bp region over the LINE-1 insertion present in a LA PRA case. Within this matrix family are five photoreceptor conserved element 1 TFBS, one cone-rod homeobox-containing transcription factor/otx-like homeobox TFBS and one pituitary homeobox 1 TFBS

To investigate whether the LINE-1 insertion may disrupt regulation of the *IMPG2* gene, *in-silico* analyses of the region surrounding the insertion were performed to search for putative regulatory sequences and promoter sequences. The Gene2Promoter tool suggested that a promoter region exists within 1.5 Kb of the upstream DNA sequence of *IMPG2.* However when using the PromoterInspector tool to predict eukaryotic Pol II promoter regions in mammalian genome sequences, no such promoter regions were predicted. Analysis of 1.5 Mb upstream and downstream of the LINE-1 insertion breakpoints using the MatInspector tool identified 1275 matches to putative transcription factor binding sites (TFBS) of which 162 were within 150 bp upstream and downstream of the LINE-1 breakpoints. Forty-three of these were associated with eye tissue including three photoreceptor conserved element 1 TFBS, one cone-rod homeobox-containing TFBS and one pituitary homeobox 1 TFBS (Supplementary Table 10). These five photoreceptor specific TFBS belong to the “vertebrates bicoid-like homeodomain transcription factor matrix family” (matrix symbol = V$BCDF) and are located within very close proximity to the LINE-1 insertion (Fig. 5). All bicoid-like homeodomain TFBS within this matrix family ‘V$BCDF’ in the dog are listed in Table 3.

**Table 3:** Genes encoding transcription factors binding to the TFBS sites of the ‘V$BCDF’ matrix family closely situated around the *IMPG2* LINE-1 insertion identified using MatInspector

Organism	Genes for Transcription Factors
Dog	CRX, DMBX1, GSC2, OTX1, OTX2, PITX1, PITX2, PITX3
Human	CRX, DMBX1, GSC, GSC2, OTX1, OTX2, PITX1, PITX2, PITX3

### Using the PRA4 DNA test to estimate allele frequencies

Validation of the LINE-1 insertion enabled the development of a DNA test to help reduce the incidence of this PRA in the LA. This form of PRA in the LA has been termed ‘PRA4’ to distinguish it from other forms of PRA described in other breeds. At the time of writing, 911 LA dogs from 22 countries have been genotyped for the *IMPG2* LINE-1 insertion displaying an allele frequency of 0.1. Genotyping data and allele frequencies are summarised in Table 4.

**Table 4:** Total number of LA dogs DNA tested for PRA4, showing genotypes and allele frequencies of the LINE-1 insertion

Genotype^a^ Cohort	PRA4 −/−	PRA4 −/+	PRA4 +/+	Total	Allele frequency
Across 22 countries	6	170	735	911	0.1
UK only	4	107	457	568	0.1

^a^The wild type allele is represented by ‘+’ and the mutant allele by ‘-‘

**Fig. 6:**
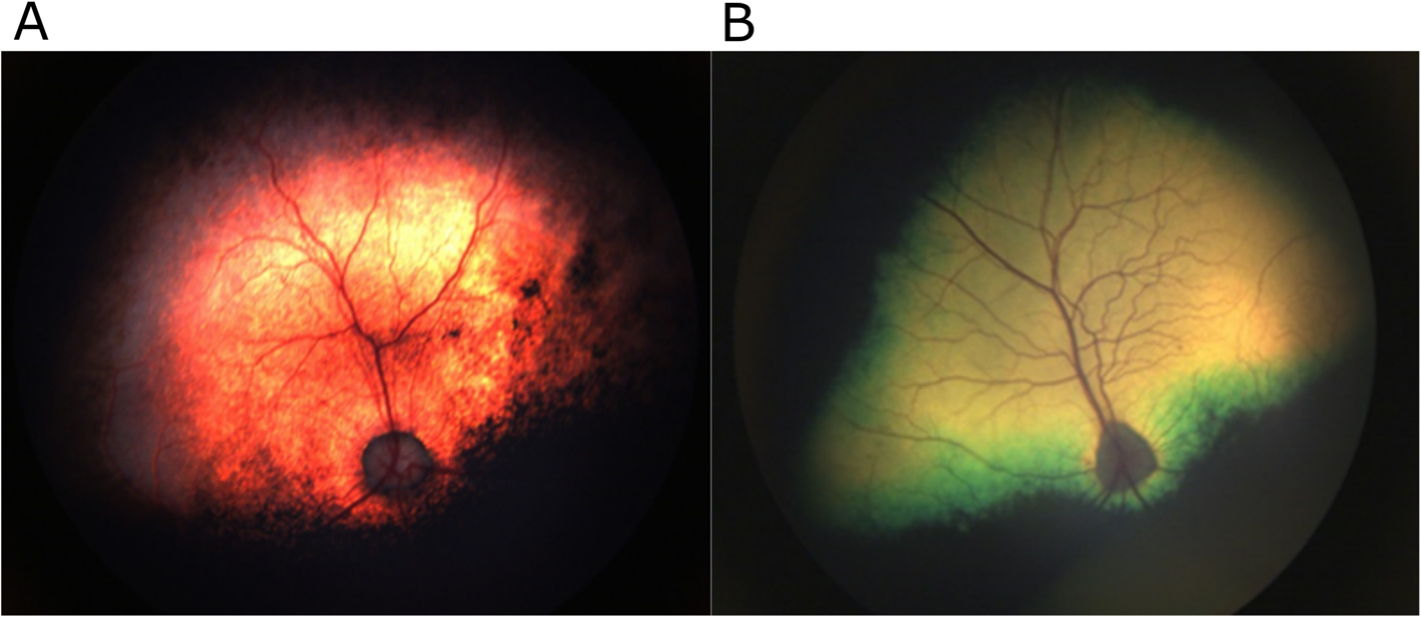
Fundus changes observed in a LA with PRA. **a** An image of the retina in the left eye of a PRA4 ^−/−^ LA dog. Bilateral tapetal hyperreflectivity was observed as well as mild vascular attenuation and changes to the optic disc colouration. **b** An image of the retina of a control dog (Giant Schnauzer dog) with a normal fundus [30]

A PRA4 homozygote (PRA4^−/−^) identified by the DNA test underwent clinical follow up and was examined by a board-certified ophthalmologist/ BVA panellist at the AHT. Upon ophthalmoscopic evaluation at the age of 2.5 years, the LA had early retinal abnormalities consistent with PRA, including tapetal hyperreflectivity, mild attenuation of blood vessels in the retina and changes to the optic disc colouration (Fig. 6).

### Sample relatedness and kinship coefficients of LA dogs to strengthen confidence in reported allele frequencies from DNA testing datasets

DNA samples from dogs selected for DNA testing are a biased sample and may not represent a random sample of the population. We wanted to test whether the allele frequency of the DNA tested population was materially different to the general population of LA. To determine if the allele frequencies reported from the PRA4 DNA test could be described as representative of the general LA population, statistical analysis was conducted on a subset of PRA4 DNA tested LA. Kinship is a determinant of the genetic similarity between two individuals and a kinship coefficient is a way of quantifying the relatedness of two individuals in an extended family or pedigree. Pairwise kinship coefficients range from 0 to 1, full siblings in outbred populations will generate a kinship coefficient of 0.25 and half siblings a coefficient of 0.125. The mean and standard deviation of pairwise kinship coefficient from the 261 AHT PRA4 tested dogs born 2009–2017 (where > 5 dogs were born in each year) were 0.094 and 0.0501, respectively. From 1500 replicates of 261 randomly sampled UK Kennel Club (KC) registered LA dogs, also born between 2009 and 2017, the mean pairwise kinship coefficient was 0.080 (sd 0.0258), range = 0.074–0.087 (Supplementary Figure 4). Both the mean and SD of these pairwise kinship coefficients in the AHT PRA4 tested sample set are significantly higher than that of the random KC registered sample sets (*P* < 0.001, confidence interval test), implying that the test sample contains some closely related individuals. Closer inspection of the distribution of pairwise kinship coefficients between the AHT sample set and the random replicate samples shows good concordance over values 0 to 0.16, but notable over-representation of kinships of the magnitude 0.161 to 0.202, and 0.421 to 0.44 in the AHT sample set (Supplementary Figure 5A-C).

A new random sample of 1000 KC registered LA dogs born 2009–17 was drawn, and pairwise kinships calculated for this group and the AHT sample (*n* = 261). From the first three principle components used in MDS plots, *n* = 16 individuals were identified as outliers (with values < 0.5 or > 99.5 percentiles). Further investigation determined that these comprised two family groups (Supplementary Figure 5D). The MDS plots show that, excluding these 16 outliers, the AHT sample set better clusters with the random KC sample (Supplementary Figure 6). This suggests that exclusion of these 16 outliers presents a population that is more representative of the general KC registered LA population. Table 5 provides the mean of pairwise kinships (relationships) between and within various groupings: Group A = the 16 outliers from the MDS plot; Group B = the AHT subset of PRA4 tested LA less the 16 outliers (*n* = 245), and Group C = the 1000 randomly sampled KC registered LA dogs born 2009–2017. The mean kinship values among Group A (*n* = 16) is 0.223; approaching that of full sibling level (0.25), indicating that they are more closely related to each other than to other dogs in Group B (0.116) and Group C (0.100). The mean kinship of Group A with Group C is higher than that of Group B with Group C (0.100 vs 0.078). In addition, the mean pairwise kinship between Group B and Group C is similar to the mean pairwise-kinship within Group C (0.078 vs 0.080). Group C is the only truly random sample. The allele frequencies of the 261 AHT PRA4 tested subset, and the same subset minus the 16 outlier dogs are reported in Table 6. Allele frequencies generated from the DNA tested population excluding the 16 outliers can be considered as representative of the general LA population.

**Table 5:** Mean kinships between and within various groupings: Group A = the 16 outliers from the MDS plot; Group B = the AHT subset of 245 PRA4 tested LA; Group C = 1000 randomly sampled KC registered LA dogs born between the years 2009–2017

	Group A (*n* = 16)	Group B (*n* = 245)	Group C (*n* = 1000)
Group A (*n* = 16)	**0.223**		
Group B (*n* = 245)	0.116	**0.091**	
Group C(*n* = 1000)	0.100	0.078	**0.080**

**Table 6:** Allele frequencies for PRA4 in the AHT subset (*n* = 261) and the AHT subset excluding the 16 outliers identified through the first three principle components used in MDS plot analysis (*n* = 245)

	Wildtype	Carrier	Mutant	Total	Allele Frequency
AHT PRA4 subset	221	38	2	261	0.081
AHT PRA4 subset excluding 16 outliers	210	33	2	245	0.076

## Discussion

In this study, a GWAS was performed to identify an interval associated with a novel autosomal recessive form of PRA in the LA. A statistically significant association was identified on CANFA33 which remained significant after correcting for multiple testing and population stratification. Analysis of a 1.3 Mb region of homozygosity on CANFA33, which was present in the GWAS PRA cases and absent in the controls, identified a LINE-1 insertion located within the predicted promoter region of *IMPG2.* PRA in the LA has not been reported in the literature; however, the disease is well recognised anecdotally in the breed and is listed on Schedule A of the UK BVA/KC/ISDS eye scheme. Pedigree analysis indicated an autosomal recessive mode of inheritance, which is common for canine PRAs.

The 1.3 Mb disease-associated region identified from GWAS analysis was homozygous in 12 of the 15 cases that passed QC. Two of the three cases that were not homozygous for the critical region were aged 6.3 years and 10 years with a BVA certificate or veterinary referral letter diagnosing PRA, respectively. The third dog had been examined by a certified veterinary ophthalmologist, with a diagnosis of suspected PRA at 11 years of age with additional clinical notes reporting that related dogs became blind due to a different eye condition; sudden acquired retinal degeneration syndrome (SARDS). A fourth PRA case, submitted after the initial GWAS, included in variant follow-up, was also found to be homozygous for the wild type allele for all four variants of interest within the critical region, including the LINE-1 insertion. This case was diagnosed with PRA at the age of 6.8 years by a Member of the Royal College of Veterinary Surgeons (MRCVS) and was unable to visit a BVA panellist or certified ophthalmologist to confirm the diagnosis. These four discordant cases are assumed to be affected with a genetically distinct form of PRA or a PRA phenocopy. A separate GWAS analysis of the three dogs that were genotyped for the GWAS but were not homozygous for the critical region was carried out using the remaining unaffected LA from the GWAS as control dogs, but revealed no suggestive loci (data not shown). Recruitment of additional PRA-affected LA dogs that are clear of the PRA4 mutation may provide scope for future studies of a second form of PRA in the breed.

Two retinal candidate genes, *IMPG2* and *CEP97*, are situated within the defined critical region on CANFA33. Both genes were manually interrogated for potential causal variants using WES data generated from LA cases and controls, which confirmed conclusions drawn from prior analysis of this WES data that no candidate exonic variants for PRA in this breed were found across the exome or within the defined critical region. This suggested that the PRA-associated variant was within a non-coding region not captured by the WES probes, including upstream promoter regions. WGS was therefore performed on one PRA-affected LA to provide a comprehensive genomic dataset. A PRA case homozygous for the critical region was chosen for WGS, to ensure it was representative of the other cases from the GWAS sharing this haplotype. The critical region was explored and a LINE-1 insertion upstream of the *IMPG2* gene was identified. Notably, no strong exonic candidate variants were identified in *CEP97*; however an intronic variant in *CEP97* was considered and genotyped in a LA cohort. Given the absence of recombination events between the LINE-1 insertion, the most associated GWAS SNP and the two intronic SNVs (in *CEP97* and *IMPG2*), the genotype frequencies were compared. Alleles illustrated in Supplementary Figure 3 show that all four variants are in close proximity to one another and indicates recombination events have occurred in two dogs between these regions. The LINE-1 insertion was considered a plausible variant as it was a better functional candidate. Although the *IMPG2* intronic SNV is as correlated as the LINE-1 insertion, predicted pathogenicity and disruption of the *IMPG2* promoter region suggested the LINE-1 insertion as the likely causal variant of PRA in these dogs.

Dog breeds exist as isolated populations each with a limited number of founders which has led to large regions throughout the genome in linkage disequilibrium (LD) [76, 77]. The significant LD that may be present in individual breeds means that it can be impossible to statistically refine the number of possible causal variants down to a single one, where regions of homozygosity and variants in LD with one another flank a disease locus. Studying additional individuals to continue to monitor genotype-phenotype concordance is important in these instances.

Mutations in *IMPG2* result in autosomal recessive RP [61] and childhood-onset rod-cone dystrophy with early macular involvement in humans [78]. Bandah-Rozenfeld et al. [61] identified seven different mutations patients with early onset RP (five nonsense mutations and a 1.8 Kb genomic deletion over exon 9) and maculopathy (one missense mutation). IMPG2 belongs to a group of glycosylated proteins called proteoglycans, which bind the large carbohydrates (glycosaminoglycans) in neural tissues. The retina consists of a neural network of layer-by-layer structures in which proteoglycans are secreted from photoreceptor cells and reside in the extracellular matrix bound to the retinal pigment epithelium (RPE) [79]. The interphotoreceptor matrix (IPM) is a unique extracellular complex surrounding retinal photoreceptor outer segments and the RPE in the fundus of the eye, and is crucial for supporting normal function of retinal photoreceptors [80, 81]. Studies have suggested that the IPM plays a role in recycling photoreceptor outer segments; in retina-RPE adhesion; the establishment of a milieu suitable for photoreceptor survival; and in the exchange of molecular products between the RPE and photoreceptor cells [80, 82, 83]. The role of *IMPG2* in retinal photoreceptors and its association with human retinal disease therefore makes it a strong candidate gene for canine retinal disorders.

Belonging to a group of transposable elements, LINE-1 elements are repetitive sequences present throughout the genome. The majority are inactive, defective elements which vary in size [84]. Full length LINE-1 elements can exceed 5 Kb in length. However, they can be truncated either at the 5′ end or further 3′ by premature polyadenylation, the addition of a polyA tail [85, 86]. Structurally they contain a 5′ untranslated region (UTR) with internal promoter activity, two open reading frames (ORFs), a 3′ UTR and a polyA tail [84]. There are a variety of mechanisms in which LINE-1 insertions can alter gene expression [87–90]. Where a transposable element is inserted upstream of a gene, transcription of that gene may be altered by (i) introducing new regulatory elements, (ii) disruption of existing cis-regulatory elements, or (iii) the introduction of alternative splice sites or start sites, the latter due to an inserted promoter sequence [84, 91].

Promoter regions are DNA sequences classically located upstream of a gene, which, along with transcription factors interacting with the promoter region, determine where transcription is initiated. Transcription factors recognize short DNA sequences, called cis-regulatory sequences, which determine which gene will be transcribed. Promoter regions upstream of genes are significant in transcriptional regulation, therefore mutations within promoter regions are commonly associated with disease [92]. In many eukaryotic genes, a conserved TATA box promoter sequence is present. However, Chen et al. [93] showed that regulatory elements excluding the TATA box were present within a 100 bp upstream of the 5′ end of *IMPG2* and were 100% conserved in human and mouse. Five regulatory elements including pineal regulatory elements (PIRE) were located within this 100 bp upstream region and four copies of the PIRE were located between 400 and 1000 bp upstream. Transcription regulation is through these additional regulatory elements [93]. Cone-rod homeobox (CRX), a cone-rod homeobox-containing transcription factor/otx-like homeobox protein, is the binding partner of PIRE, inducing transactivation of a PIRE reporter construct [94] and is expressed in retinal photoreceptor cells. In the present study, CRX is also one of the genes encoding transcription factors represented by the ‘V$BCDF’ matrix family in the in silico prediction tool MatInspector [55]. The presence of PIRE is thus likely to be important in controlling the expression of *IMPG2* in photoreceptors in the retina [93]. Moreover, these TFBS elements may be disrupted by the insertion of the LINE-1 sequence in PRA-affected LA dogs, which in turn may impact IMPG2 transcription and protein function. An example of a LINE-1 element within a promoter region associated with disease was described by Davidson et al. in human patients with autosomal dominant corneal endothelial dystrophies [95]. Four mutations within a conserved promoter region of the *OVOL2* gene were suggested to alter predicted TFBS. This dysregulated *OVOL2* expression impacted the function of downstream genes and pathways, including transcriptional regulation. Furthermore, transposable elements located in non-exonic regions have been associated with inherited retinal diseases in dogs. An intronic LINE-1 insertion in a putative regulatory region of the *MERTK* gene was found to be associated with a retinopathy in Swedish Vallhund dogs [96]. In addition, an intronic short interspersed nuclear element (SINE) insertion near the splice acceptor site of *FAM161A* was identified in PRA-affected Tibetan Spaniel and Tibetan Terrier dogs [22]. In order to determine the direct impact of a transposable element on gene regulation or expression, as in studies aforementioned, blood or tissue from affected individuals is required. As no retinal or CRX expressing tissue was available from any cases in the current study, a luciferase assay was attempted using canine skin cells, a cell line available for immediate use, where expression of the *IMPG2* gene was confirmed by qPCR. However, due to the absence of CRX expression in this cell type, this luciferase assay was unsuccessful. Since the hypothesis that transcription was disrupted by the LINE-1 element could not be tested either by luciferase assay or RNA or protein expression analysis, the effect of the LINE-1 insertion observed in this study on the *IMPG2* gene can only be speculated. The lack of tissue from affected dogs is a common issue in canine PRA investigations, where the majority of affected dogs do not require enucleation as a result of the disease.

DNA tests are used by dog owners and breeders as a tool to prevent affected offspring being born with a particular inherited condition. The outcome of this study has been the development of a DNA test, termed PRA4, to enable LA dogs to be tested for this form of PRA. Research by Lewis and Mellersh has shown that a DNA tested population is a biased population [97]. Therefore, as this is a new DNA test, and as allele frequencies generated from the DNA test may not be applicable to the general LA dog population, statistical analyses were performed. It was unknown how representative of the general population the PRA4 DNA tested population is, and if many individuals are closely related then allele frequencies and carrier rates can be skewed. Using a subset of 261 PRA4 tested LA dogs, statistical analysis was performed to compare relatedness across individuals, and identify dogs that were closely related in the DNA tested subset in order to try and provide a less skewed frequency statistic. Sixteen outliers were apparent from the MDS plot of kinship coefficient. Pedigree information for these 16 individuals revealed they belonged to two families and these dogs were therefore closely related. The mean kinship values suggest that, although there are some closely related individuals in the AHT PRA4 subset, generally the AHT tested sample (when discounting the 16 outliers) was representative of the wider population. Allele frequencies generated including and excluding these outliers also support this and provide confidence in allele frequencies generated from a DNA tested population, particularly in the period of time immediately following the availability of a new DNA test. The recently estimated mutant allele frequency of 0.1, generated from the 911 DNA tested LA during 2 years of use of a DNA test based on this work, indicates that 1 in 100 dogs are likely to be affected with this form of PRA, and an 18% carrier frequency within the LA population. Although this population will likely include closely related dogs, this value is well within the range presented by estimated allele frequencies of other recessive conditions in canine studies [13, 19, 21].

Clinical follow up of one PRA4 ^−/−^ individual has provided some evidence that the age of onset in the breed is variable, where clinical signs of retinal changes can be present from as early as 2.5 years of age. Where provided with sample clinical information, the age of onset of PRA cases homozygous for the disease-associated haplotype from the GWAS varied, ranging from aged 1.75–10 years. The owner of the PRA4^−/−^ individual had not noticed behavioural changes or signs that the dog’s vision was deteriorating, suggesting these were early signs of the slowly progressive disease that were only apparent upon ophthalmoscopic examination. Continual annual checks of this dog and other PRA4 ^−/−^ LA dogs will help describe the rate of progression of PRA in this breed.

## Conclusions

We have identified a LINE-1 insertion upstream of the *IMPG2* gene that strongly segregates with PRA in LA dogs. Extensive genotyping of this variant in multiple breeds strongly suggested that the LINE-1 insertion is private to the LA and was only present in PRA-affected dogs. Utilisation of the PRA4 DNA test will, over time, help reduce the frequency and incidence of this mutation in the LA breed.

## Supplementary information

**Additional file 1: Supplementary Table 1.** (A) PCR primers used for sequencing amplicons of 18 known canine retinal mutations in the PRA-affected LA sent for WGS. (B) PCR primers used for genotyping seven known canine retinal mutations in the PRA-affected LA sent for WGS (by PCR followed by amplified fragment length polymorphism (AFLP) analysis or by visualisation of PCR product on an agarose gel). **Supplementary Table 2.** Multiplex PCR amplification using pooled primers. **Supplementary Table 3.** Thermal cycling conditions for multiplex PCR amplification using pooled primers. **Supplementary Table 4.** Reaction for *IMPG2* LINE-1 insertion amplification for size determination. **Supplementary Table 5.** Thermal cycling conditions to amplify *IMPG2* LINE-1 insertion. **Supplementary Table 6.** Primer sequences to amplify candidate variant regions. **Supplementary Table 7.** Amplification of *IMPG2* LINE-1 insertion for amplified fragment length polymorphism analysis. **Supplementary Table 8.** Thermal cycling conditions for amplification of *IMPG2* LINE-1 insertion for amplified fragment length polymorphism analysis. **Supplementary Table 9.** Breed names for 447 dogs of 123 breeds that were screened for the *IMPG2* LINE-1 insertion. **Supplementary Table 10.** Forty-two transcription factor binding site predictions from MatInspector in eye tissue within 150 bp upstream and downstream of the *IMPG2* LINE-1 breakpoints. Five of these are bicoid-like homeodomain transcription factors (highlighted in orange) and are specific to photoreceptor cells in the retina. **Supplementary Figure 1.** A multi-dimensional scaling plot to determine relatedness between the case and control sample sets showed a similar distribution of 15 cases and 27 controls analysed in the GWAS. **Supplementary Figure 2.** (A) The quantile-quantile (Q-Q) plot of the expected and observed –log^10^ *p* values generated from PLINK derived a genomic inflation factor, lambda (λ) =1.36. (B) The Q-Q plot after correcting for population stratification using EMMAX showed a decreased inflation factor, λ =1.02. **Supplementary Figure 3.** A schematic diagram showing genotypes for four variants across 18 PRA-affected (A1–18) LA and 41 PRA-unaffected (C1–41) LA: homozygous alternate allele (coloured pink), homozygous wild type/reference allele (coloured yellow) or heterozygous (coloured pink and yellow). **Supplementary Figure 4.** The distribution of the random sample sets mean pairwise kinships (blue histogram), and the AHT PRA4 DNA tested sample set (red dotted line). **Supplementary Figure 5.** (A-C) Histograms showing the proportion of pairwise relationships across the random sample sets and the AHT PRA4 DNA tested subset; (D) Pedigree drawing of the 16 outliers belonging to two distinct families: circle = female, square = male, diamond = unknown, shaded diamond = not included in our data set. **Supplementary Figure 6.** (A) Multi-dimensional scaling plot to determine relatedness within each sample set. Red points represent the 261 AHT PRA4 tested samples, blue points represent 1000 randomly selected KC registered dogs born 2009–2017); (B) zoomed in on central cluster in (A) showing the main body of the AHT sample set (red) is representative of a random sample (blue).

## Data Availability

The datasets generated and/or analysed during the current study are available in the European Nucleotide Archive (ENA) repository, https://www.ebi.ac.uk/ena/data/view/PRJEB35025; https://www.ebi.ac.uk/ena/data/view/ERS3907556; https://www.ebi.ac.uk/ena/data/view/ERR3609666.

## Abbreviations

AFLP: Amplified fragment length polymorphism
AHT: Animal Health Trust
bp: Base pair
BVA/KC/ISDS: British Veterinary Association/ Kennel Club/ International Sheep Dog Society
CANFA: Canine chromosome
CEP97: Centrosomal protein 97
CRX: Cone-rod homeobox
EMMAX: Efficient Mixed-Model Association eXpedited
ERG: Electroretinogram
ECVO: European College of Veterinary Ophthalmologists
GWAS: Genome-wide association study
indel: Insertion/deletion variants
IPM: Interphotoreceptor matrix
IMPG2: Interphotoreceptor matrix proteoglycan 2
KC: The Kennel Club
LA: Lhasa Apso
LD: Linkage disequilibrium
LINE-1: Long interspersed element-1
Mb: Megabase
μL: Microliter
MDS: Multi-dimensional scaling
NGS: Nanograms
NGS: Next-generation sequencing
ONL: Outer nuclear layer
PIRE: Pineal regulatory elements
PRA: Progressive retinal atrophy
QC: Quality-control
Q-Q: Quantile-quantile
qPCR: Quantitative PCR
RPE: Retinal pigment epithelium
RP: Retinitis pigmentosa
SINE: Short interspersed nuclear element
SNP: Single nucleotide polymorphism
SNV: Single nucleotide variant
TFBS: Transcription factor binding sites
UTR: Untranslated region
VCF: Variant Call Format
VEP: Variant Effect Predictor
WES: Whole-exome sequencing
WGS: Whole-genome sequencing
