# Supplementary material for: A LINE-1 insertion situated in the promoter of IMPG2 is associated with autosomal recessive progressive retinal atrophy in Lhasa Apso dogs
Source: BMC Genet. 2020 Sep 7;21:100. doi: 10.1186/s12863-020-00911-w (PMC7487703; doi:10.1186/s12863-020-00911-w)
Supplement: Supplementary file 1 — Additional file 1: Supplementary Table 1. (A) PCR primers used for sequencing amplicons of 18 known canine retinal mutations in the PRA-affected LA sent for WGS. (B) PCR primers used for genotyping seven known canine retinal mutations in the PRA-affected LA sent for WGS (by PCR followed by amplified fragment length polymorphism (AFLP) analysis or by visualisation of PCR product on an agarose gel). Supplementary Table 2. Multiplex PCR amplification using pooled primers. Supplementary Table 3. Thermal cycling conditions for multiplex PCR amplification using pooled primers. Supplementary Table 4. Reaction for IMPG2 LINE-1 insertion amplification for size determination. Supplementary Table 5. Thermal cycling conditions to amplify IMPG2 LINE-1 insertion. Supplementary Table 6. Primer sequences to amplify candidate variant regions. Supplementary Table 7. Amplification of IMPG2 LINE-1 insertion for amplified fragment length polymorphism analysis. Supplementary Table 8. Thermal cycling conditions for amplification of IMPG2 LINE-1 insertion for amplified fragment length polymorphism analysis. Supplementary Table 9. Breed names for 447 dogs of 123 breeds that were screened for the IMPG2 LINE-1 insertion. Supplementary Table 10. Forty-two transcription factor binding site predictions from MatInspector in eye tissue within 150 bp upstream and downstream of the IMPG2 LINE-1 breakpoints. Five of these are bicoid-like homeodomain transcription factors (highlighted in orange) and are specific to photoreceptor cells in the retina. Supplementary Figure 1. A multi-dimensional scaling plot to determine relatedness between the case and control sample sets showed a similar distribution of 15 cases and 27 controls analysed in the GWAS. Supplementary Figure 2. (A) The quantile-quantile (Q-Q) plot of the expected and observed –log10 p values generated from PLINK derived a genomic inflation factor, lambda (λ) =1.36. (B) The Q-Q plot after correcting for population stratification using EMMAX [file 12863_2020_911_MOESM1_ESM.docx]

**Supplementary Table 1: (A)** PCR primers used for sequencing amplicons of 18 known canine retinal mutations in the PRA-affected LA sent for WGS. **(B)** PCR primers used for genotyping seven known canine retinal mutations in the PRA-affected LA sent for WGS (by PCR followed by amplified fragment length polymorphism (AFLP) analysis or by visualisation of PCR product on an agarose gel).

| **Form of retinal degeneration** | **Locus or**  **Abbreviation** | **Gene**  **involved** | | **Primer**  **sequences** | **References** |
| --- | --- | --- | --- | --- | --- |
| **(A) Pooled for multiplex PCR for amplicon sequencing** | | | | | |
| Cone degeneration | CD | **CNGB3** | | F-CCCACTTACCATTATGTCTCCTC; R-TCAAACACCAGACAACACACA | [1, 2] |
| Canine achromatopsia | ACHM | **CNGA3** | | SNP_F-AGTGGGCGTCCTAATCTTTG; SNP_R-CGGAATTGCATGTACTGCTT | [3] |
| Canine achromatopsia | ACHM | **CNGA3** | | del_F-CCTGATCGACGAGGACGTG; del_R-CGTTGTACTCGGCCAGGAG | [3] |
| Congenital stationary night blindness/retinal dystrophy | CSNB | **RPE65** | | F-GACAATGCCCTTGTTAACGTC; R-AGCATTTCGTCCTACCTGCTT | [4], [5] |
| Canine multifocal retinopathy | CMR1 | **BEST1/ VMD2** | | F-CGTCACCTACTCAAGCCAAGT; R-TAGCTGAGCAGGAAGATGAGG | [6] |
| Canine multifocal retinopathy | CMR2 | **BEST1/ VMD2** | | F-GGCCCTCCCCATCCACTG; R-CAGGGCACCCAGAACGTGT | [6] |
| Canine multifocal retinopathy | CMR3 | **BEST1/ VMD2** | | F-AAGGCCTGGGAGATTAAGGAG; R-CTGACTGTCCAGATGGGAAGA | [7] |
| Generalised PRA | gPRA | **CCDC66** | | F-GCTCATACTGCTTAGATGTCTCTTG; R-CAATACTTCCAATCAGCCAAA | [8], [9] |
| Progressive retinal atrophy | PRA | **CNGB1** | | F-CCACCCAGGCTCAGCAG; R-TCAGGCAGCCCACCAAT | [10] |
| Photoreceptor dysplasia | PD | **PDC** | | F-TGTCTATTCCCAGATGAGCATT; R-AAACTCAGCTTCTGGTGCATATC | [11] |
| Progressive rod-cone degeneration | PRCD | **PRCD** | | F-TTTCTCCTGCAGACTCTGTCC; R-CAGCTTCTCACGGTTGGA | [12] |
| Autosomal dominant PRA | ADPRA | **RHO** | | F-ACACCCGTCTTGTTGGAGAA; R-CCTCAGCAGCACTCTTAGGAC | [13] |
| Progressive retinal atrophy | GR_PRA1 | **SLC4A3** | | F-CTGCCCAGGTGAGTGCTAGA; R-CACTCCCGTTCAGCTCCAG | [14] |
| Progressive retinal atrophy | GR_PRA2 | **TTC8** | | F-TGGACTTGGCTGCCCTTT; R-CCATGTCTAAGCCCTTCACAA | [15] |
| Progressive retinal atrophy | PRA | **CNGA1** | | F-GCAATCGAAGAACAGCCAAT; R-GCTTTCCCTTCTCTTCTAGCAT | [16] |
| Rod-cone dystrophy | RCD1 | **PDE6B** | | F-GAAGAGATCCTGCCCATGTT; R-TGTCCTCTTGTTGCTGCTTCT | [17, 18] |
| Rod-cone dystrophy | RCD1a | **PDE6B** | | F-GAAGAGATCCTGCCCATGTT; R-TGTCCTCTTGTTGCTGCTTCT | [19] |
| Rod-cone dystrophy | RCD3 | **PDE6A** | | F-CAGGACTGGGTGAGGATGATA; R-ACTTGAAATACAGGGCGAGGT | [20] |
| **(B) Amplified fragment length polymorphism (AFLP) analysis** | | | | | |
| Cone-rod dystrophy | CORD1 | **RPGRIP1** | 6FAM-GAAGAGCACATGTTGGTGAAGG; R- TGAGCTTTGTTTGCCTTTGG | | [21], [22] |
| Cone-rod dystrophy (early onset) | EORD | **MAP9** | 6FAM-GTCGATGGAGGTCTCCGTAT; R- AACCTGGACCATGGGCAATA | | [23] |
| Rod-cone degeneration | RCD4 | **C2ORF71** | 6FAM-CCGAGTGCTCCCTCTGTG ; R- GGCTGCAGGCCTCGTC | | [24] |
| Progressive retinal atrophy | PRA3 | **FAM161A** | Aff_F- GGATCCCTTTATTTGATTTTAGAAAG;  Nor_F- TCCCTTCCTTTTATTTGATTTTAGAAAG;  R- 6FAM-CAACAAACACAACCTGAGCAA | | [25] |
| Agarose gel electrophoresis | | | | | |
| Cone-rod dystrophy | CRD3 | **ADAM9** | Nor_F- AAGTACCTACCCTCCCGTTCA;  Nor_R- GTAGGATGGAGGTCGAAGAGG;  Aff-F- TTGGAACACCTAAATGCCTTG;  Aff-R- GTAACCCCAGCCAACACAGTA | | [26] |
| Cone-rod dystrophy | CRD | **NPHP4** | F- AGTTCCTTCAGTTACGTCAACCAT; R- CTTCCAACACACAGACAGTACTCC | | [27] |
| Early retinal degeneration | ERD | **STK38L** | F- CAGCCGCACCTAAAATGTAAA; R- CTTTATTCTTGGGTGAACTCCA | | [28], [29] |

Supplementary Table 2: Multiplex PCR amplification using pooled primers.

| Component | Volume/reaction (μL) | Final concentration |
| --- | --- | --- |
| Primer mix | 5 | 0.2 mM |
| dNTPs (1.5mM) | 2.27 | 0.2 mM |
| HotStarTaq Plus 10x PCR buffer | 1.70 | 1X |
| MgCl_2_ (25X) | 1.02 | 1.5 mM |
| HotStarTaq Plus Polymerase (5 units/μL) | 0.30 | 0.09 units/μL |
| Water | 4.71 |  |
| Total (μL) | 15 |  |

Supplementary Table 3: Thermal cycling conditions for multiplex PCR amplification using pooled primers.

| Temperature (°C) | Time |  |
| --- | --- | --- |
| 95 | 10 minutes |  |
| 95 | 15 seconds | 25 cycles |
| 60 | 2 minutes |  |
| 60 | 30 minutes |  |
| 12 | Hold |  |

Supplementary Table 4: Reaction for *IMPG2* LINE-1 insertion amplification for size determination.

| Component | Volume/reaction (μL) | Final concentration |
| --- | --- | --- |
| dNTPs (1.5 mM) | 4 | 0.2 mM |
| PrimeStar GXL 5X buffer | 10 | 1X |
| Forward Primer 1 (20 μM) | 0.75 | 0.3 μM |
| Forward Primer 2 (20 μM) | 0.75 | 0.3 μM |
| Reverse Primer (20 μM) | 0.75 | 0.3 μM |
| PrimeStar GXL polymerase (1.25 units/μL) | 1 | 1.25 units/μL |
| Water | 30.75 |  |
| Total (μL) | 48 |  |

Supplementary Table 5: Thermal cycling conditions to amplify *IMPG2* LINE-1 insertion.

| Temperature (°C) | Time |  |
| --- | --- | --- |
| 98 | 10 seconds | 30 cycles |
| 60 | 15 seconds |  |
| 68 | 8 minutes |  |
| 12 | Hold |  |

Supplementary Table 6: Primer sequences to amplify candidate variant regions.

| Variant | Primers |
| --- | --- |
| *IMPG2* LINE-1 | Forward_normal: 5’-GATTTCAGGAAAGAAGTGAGAGG-3’  Forward_affected: 5’-TCTGAAGAAAGGAAAGGACACCAT-3’  Reverse: 5’-/56-FAM/ACCCAGAGAAATCTTCCCAAA-3’ |
| *IMPG2* intronic SNV (CANFA 33: 7717298) | qPCR1 primer 1: 5’-GGGACAAAGGAAACAGGTATCA-3’  qPCR1 primer 2: 5’-GAAGGAGAAGCAGGCTCCAT-3’  qPCR1 probe 1: HEX-CCCAGTGG/Zen/CTCAGCGGTTTAG-IBFQ  qPCR1 probe 2: FAM-CCCAGTGGC/Zen/TCAGCAGTTTAG-IBFQ |
| SNP BICF2G630247609 | Forward: 5’- TGAAGGTAAGTGTGTCATTTCTGA-3’  Reverse: 5’- GTTAACCTTAACTACAATGACCAGTTT-3’ |
| *CEP97* intronic SNV (CANFA33: 8044097) | Forward: 5’-CAATGAAATGTTAGCAGGGCTAC -3’  Reverse: 5’- GGAGAATCGGAGAATCCAGA -3 |

Supplementary Table 7: Amplification of *IMPG2* LINE-1 insertion for amplified fragment length polymorphism analysis.

| Component | Volume/reaction (μL) | Final concentration |
| --- | --- | --- |
| dNTPs (1.5 mM) | 1.6 | 0.2 mM |
| HotStarTaq Plus 10X PCR buffer | 1.2 | 1X |
| Forward Primer 1 (10 μM) | 0.22 | 0.18 μM |
| Forward Primer 2 (10 μM) | 0.11 | 0.09 μM |
| Reverse Primer (10 μM) | 0.22 | 0.18 μM |
| HotStarTaq Plus Polymerase (5 units/μL) | 0.24 | 0.1 units/μL |
| Water | 6.42 |  |
| Total (μL) | 10 |  |

Supplementary Table 8: Thermal cycling conditions for amplification of *IMPG2* LINE-1 insertion for amplified fragment length polymorphism analysis.

| Temperature (°C) | Time |  |
| --- | --- | --- |
| 95 | 5 minutes |  |
| 95 | 30 seconds | 35 cycles |
| 60 | 30 seconds |  |
| 72 | 30 seconds |  |
| 72 | 5 minutes |  |
| 12 | Hold |  |

Supplementary Table 9: Breed names for 447 dogs of 123 breeds that were screened for the *IMPG2* LINE-1 insertion

| Breed | Number of individuals genotyped |
| --- | --- |
| American Bulldog | 3 |
| Australian Cattle Dog | 3 |
| American Cocker Spaniel | 3 |
| Affenpinscher | 3 |
| Afghan Hound | 3 |
| Australian Kelpie | 3 |
| Alaskan Malamute | 3 |
| Australian Shepherd | 3 |
| Airedale Terrier | 3 |
| Boxer | 3 |
| Basset Hound | 3 |
| Border Collie | 3 |
| Bulldog | 3 |
| Beagle | 3 |
| Bearded Collie | 3 |
| Basset Fauve De Bretagne | 3 |
| Petit Basset Griffon Vendéen | 3 |
| Bloodhound | 3 |
| Border Terrier | 3 |
| Borzoi | 3 |
| Berger Picard (Picardy sheepdog) | 3 |
| Brittany Spaniel | 3 |
| Belgian Shepherd Dog (Tervueren) | 2 |
| Belgian Shepherd Dog (Groenendael) | 1 |
| Boston Terrier | 3 |
| Bull Terrier | 3 |
| Chesapeake Bay Retriever | 3 |
| Curly Coated Retriever | 3 |
| Cesky Terrier | 3 |
| Chow Chow | 3 |
| Cavalier King Charles Spaniel | 3 |
| Cocker Spaniel | 3 |
| Catalan Sheepdog | 3 |
| Doberman | 3 |
| Dandie Dinmont | 3 |
| Dalmatian | 3 |
| English Setter | 3 |
| English Springer Spaniel | 3 |
| French Bull Dog | 3 |
| Flat Coated Retriever | 3 |
| Finnish Lapphund | 3 |
| Field Spaniel | 3 |
| Greyhound | 3 |
| Grand Basset Griffon Vendéen | 3 |
| Great Dane | 3 |
| Glen of Imaal Terrier | 3 |
| Gordon Setter | 3 |
| German Pinscher | 3 |
| Golden Retriever | 3 |
| Giant Schnauzer | 3 |
| German Shepherd Dog | 3 |
| Greater Swiss Mountain Dog | 3 |
| German Spitz (Klein) | 3 |
| German Wire-haired Pointer | 3 |
| Havanese | 3 |
| Hovawart | 3 |
| Icelandic Sheepdog | 3 |
| Italian Greyhound | 3 |
| Irish Red and White Setter | 3 |
| Irish Setter | 3 |
| Italian Spinone | 3 |
| Irish Water Spaniel | 3 |
| Japanese Akita | 3 |
| Jack Russell Terrier | 3 |
| Japanese Shiba Inu | 3 |
| Keeshond | 3 |
| Lhasa Apso | 63 |
| Leonberger | 3 |
| Lancashire Heeler | 3 |
| Long-Haired Dachshund (Standard) | 3 |
| Large Münsterländer | 3 |
| Labrador Retriever | 3 |
| Lagotto Romagnolo | 2 |
| Lakeland Terrier | 3 |
| Miniature Bull Terrier | 3 |
| Miniature Long-Haired Dachshund | 3 |
| Miniature Schnauzer | 3 |
| Minature Smooth-Haired Dachshund | 3 |
| Manchester Terrier | 1 |
| Miniature Wire-Haired Dachshund | 3 |
| Norwegian Buhund | 3 |
| Norwegian Elkhound | 3 |
| Northern Inuit | 3 |
| Norfolk Terrier | 3 |
| Nova Scotia Duck Tolling Retriever | 3 |
| Norwich Terrier | 3 |
| Old English Mastiff | 3 |
| Old English Sheepdog | 3 |
| Otterhound | 3 |
| Pharaoh Hound | 3 |
| Polish Lowland Sheepdog | 3 |
| Parson Russell Terrier | 3 |
| Pug | 2 |
| Pembroke Welsh Corgi | 3 |
| Rough Collie | 3 |
| Rhodesian Ridgeback | 3 |
| Standard Schnauzer | 3 |
| Saluki | 3 |
| Samoyed | 3 |
| Staffordshire Bull Terrier | 3 |
| Scottish Deerhound | 3 |
| Scottish Terrier | 3 |
| Soft-coated Wheaten Terrier | 3 |
| Smooth Haired Dachshund (Standard) | 3 |
| Shar Pei | 3 |
| Siberian Husky | 2 |
| Standard Poodle | 3 |
| Shetland Sheepdog | 3 |
| Skye Terrier | 3 |
| Saint Bernard | 3 |
| Shih Tzu | 20 |
| Swedish Vallhund | 3 |
| Tibetan Spaniel | 10 |
| Tibetan Terrier | 5 |
| Utonagan | 3 |
| Vizsla (Smooth coat) | 3 |
| Vizsla (Wire-haired) | 3 |
| Weimaraner | 3 |
| Wire-Haired Dachshund (Standard) | 3 |
| West Highland White Terrier | 3 |
| Whippet | 3 |
| Working Sheepdog | 3 |
| Welsh Springer Spaniel | 3 |

Supplementary Table 10: Forty-two transcription factor binding site predictions from MatInspector in eye tissue within 150 bp upstream and downstream of the *IMPG2* LINE-1 breakpoints. Five of these are bicoid-like homeodomain transcription factors (highlighted in orange) and are specific to photoreceptor cells in the retina.

| **MatInspector Matrix Family** | **Detailed Family Information** | **Detailed Matrix Information** | **Sequence** |
| --- | --- | --- | --- |
| V$HBOX | Homeobox transcription factors | Homeobox protein engrailed (en-1) | tctttgccTTTAactggca |
| V$BCDF | Bicoid-like homeodomain transcription factors | Pituitary Homeobox 1 (Ptx1, Pitx-1) | ccagCTAAgctctttat |
| V$BCDF | Bicoid-like homeodomain transcription factors | Cone-rod homeobox-containing transcription factor / otx-like homeobox gene | tattcTAATctagccag |
| V$CART | Cart-1 (cartilage homeoprotein 1) | Binding site for S8 type homeodomains | taagcTAATtagaacagatgg |
| V$BCDF | Bicoid-like homeodomain transcription factors | Photoreceptor conserved element 1 | taagcTAATtagaacag |
| V$BRNF | Brn POU domain factors | POU class 3 homeobox 2 (POU3F2), OTF7 | tctgttcTAATtagcttat |
| V$HBOX | Homeobox transcription factors | Homeodomain transcription factor Gsh-2 | ataagcTAATtagaacaga |
| V$LHXF | Lim homeodomain factors | LIM homeobox 8 | catctgttctAATTagcttatct |
| V$PAXH | PAX homeodomain binding sites | Paired box 4, homeodomain binding site | aagctAATTagaaca |
| V$BCDF | Bicoid-like homeodomain transcription factors | Photoreceptor conserved element 1 | tgttcTAATtagcttat |
| V$BRNF | Brn POU domain factors | POU class 3 homeobox 2 (POU3F2), OTF7 | gataagcTAATtagaacag |
| V$HBOX | Homeobox transcription factors | Homeodomain transcription factor Gsh-2 | ctgttcTAATtagcttatc |
| V$LHXF | Lim homeodomain factors | LIM homeobox 6 | gagataagcTAATtagaacagat |
| V$PAXH | PAX homeodomain binding sites | Paired box 4, homeodomain binding site | gttctAATTagctta |
| V$CART | Cart-1 (cartilage homeoprotein 1) | Binding site for S8 type homeodomains | tgttcTAATtagcttatctct |
| V$LHXF | Lim homeodomain factors | LIM-homeodomain transcription factor LHX3 | ggaagagataagcTAATtagaac |
| V$PAX6 | PAX-4/PAX-6 paired domain binding sites | PAX6 paired domain and homeodomain are required for binding to this site | tcctgaaatCCAGcacagc |
| V$FKHD | Fork head domain factors | Fork head homologous X binds DNA with a dual sequence specificity (FHXA and FHXB) | cctttcAAAAtacctct |
| V$NR2F | Nuclear receptor subfamily 2 factors | Chicken ovalbumin upstream promoter transcription factor 2, NR2F2 homodimer, DR1 sites | gaggtattttgaaaGGACaccattt |
| V$CART | Cart-1 (cartilage homeoprotein 1) | Prophet of Pit 1, PROP paired-like homeobox 1 | tagcttAAATaaaaatggtgt |
| V$LHXF | Lim homeodomain factors | LIM homeobox transcription factor 1, beta | caccatttttATTTaagctaatt |
| V$LHXF | Lim homeodomain factors | LIM homeobox transcription factor 1, alpha | aaattagcttAAATaaaaatggt |
| V$LHXF | Lim homeodomain factors | LIM-homeodomain transcription factor LHX3 | tttttatttaagcTAATttataa |
| V$CART | Cart-1 (cartilage homeoprotein 1) | Aristaless-like homeobox 3 | attataAATTagcttaaataa |
| V$HBOX | Homeobox transcription factors | Engrailed homeobox 2 | cattataAATTagcttaaa |
| V$LHXF | Lim homeodomain factors | LIM homeobox 6 | tatttaagcTAATttataatggt |
| V$PAXH | PAX homeodomain binding sites | Paired box 6, homeodomain binding site | ttataAATTagctta |
| V$BCDF | Bicoid-like homeodomain transcription factors | Photoreceptor conserved element 1 | taagcTAATttataatg |
| V$BRNF | Brn POU domain factors | POU class 4 homeobox 3 (POU4F3), BRN3C | ccattatAAATtagcttaa |
| V$HBOX | Homeobox transcription factors | Homeodomain transcription factor Gsh-2 | ttaagcTAATttataatgg |
| V$LHXF | Lim homeodomain factors | LIM homeobox 1 | aaccattataAATTagcttaaat |
| V$CART | Cart-1 (cartilage homeoprotein 1) | Phox2a (ARIX) and Phox2b | taagcTAATttataatggttt |
| V$BRNF | Brn POU domain factors | Brn-3, POU-IV protein class | agctaatttATAAtggttt |
| V$FKHD | Fork head domain factors | HNF-3/Fkh Homolog 1 (FOXQ1) | caaacaTAAActcttaa |
| V$PAX6 | PAX-4/PAX-6 paired domain binding sites | Pax-6 paired domain binding site | cataaACTCttaaatgtta |
| V$FKHD | Fork head domain factors | Fork head homologous X binds DNA with a dual sequence specificity (FHXA and FHXB) | tttcctATAAtaatttg |
| V$BRNF | Brn POU domain factors | Brn-3, POU-IV protein class | tttcctaTAATaatttgtt |
| V$BRNF | Brn POU domain factors | Brn-3, POU-IV protein class | cctataaTAATttgttact |
| V$HBOX | Homeobox transcription factors | Engrailed homeobox 1 | agtaacaAATTattatagg |
| V$PAXH | PAX homeodomain binding sites | Paired box 6, homeodomain binding site | taacaAATTattata |
| V$BRNF | Brn POU domain factors | Brn-3, POU-IV protein class | cagtaacaaATTAttatag |
| V$CART | Cart-1 (cartilage homeoprotein 1) | HESX homeobox 1, dimeric binding site | tataaTAATttgttactggaa |


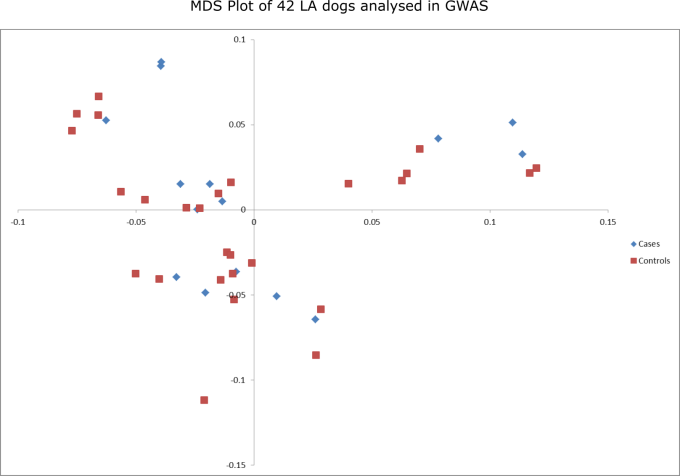


Supplementary Figure 1: A multi-dimensional scaling plot to determine relatedness between the case and control sample sets showed a similar distribution of 15 cases and 27 controls analysed in the GWAS.

**
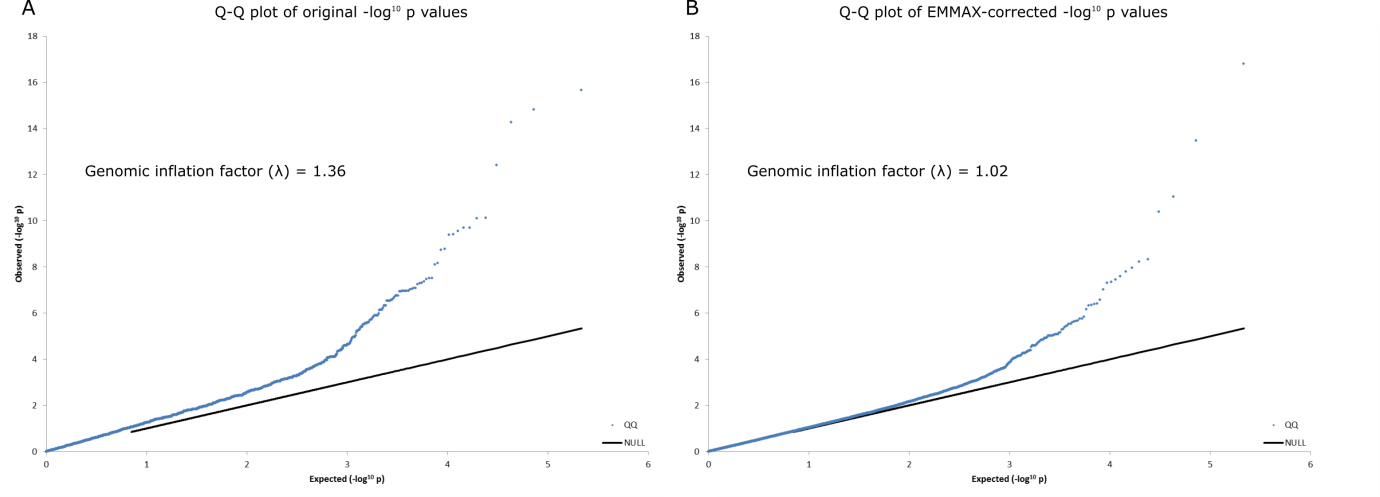
Supplementary Figure 2:** (A) The quantile-quantile (Q-Q) plot of the expected and observed –log^10^ p values generated from PLINK derived a genomic inflation factor, lambda (λ) =1.36. (B) The Q-Q plot after correcting for population stratification using EMMAX showed a decreased inflation factor, λ =1.02.


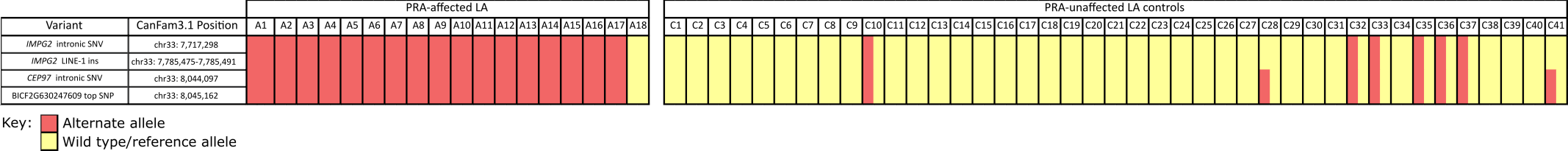


Supplementary Figure 3: A schematic diagram showing genotypes for four variants across 18 PRA-affected (A1-18) LA and 41 PRA-unaffected (C1-41) LA: homozygous alternate allele (coloured pink), homozygous wild type/reference allele (coloured yellow) or heterozygous (coloured pink and yellow).


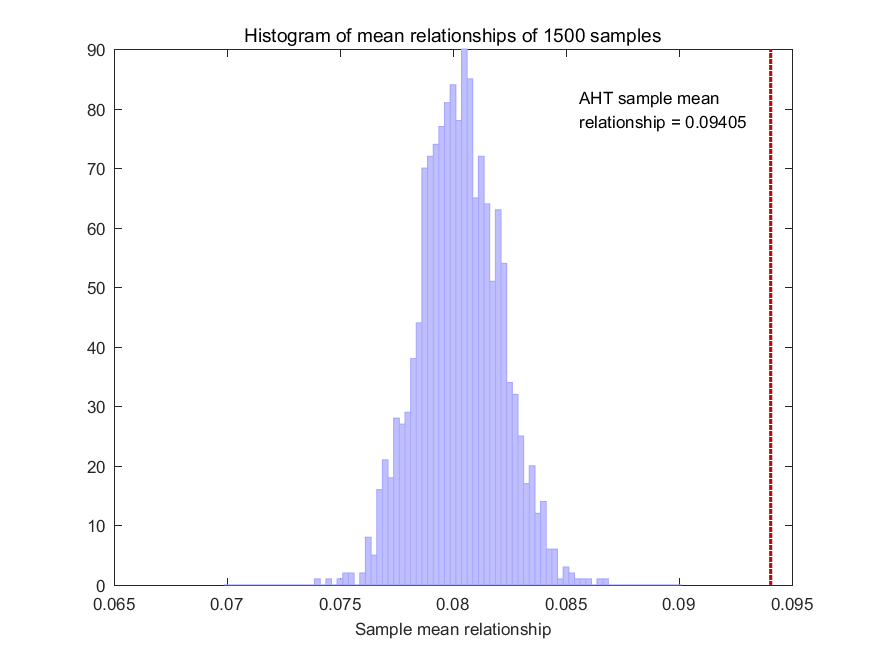


Supplementary Figure 4: The distribution of the random sample sets mean pairwise kinships (blue histogram), and the AHT PRA4 DNA tested sample set (red dotted line).


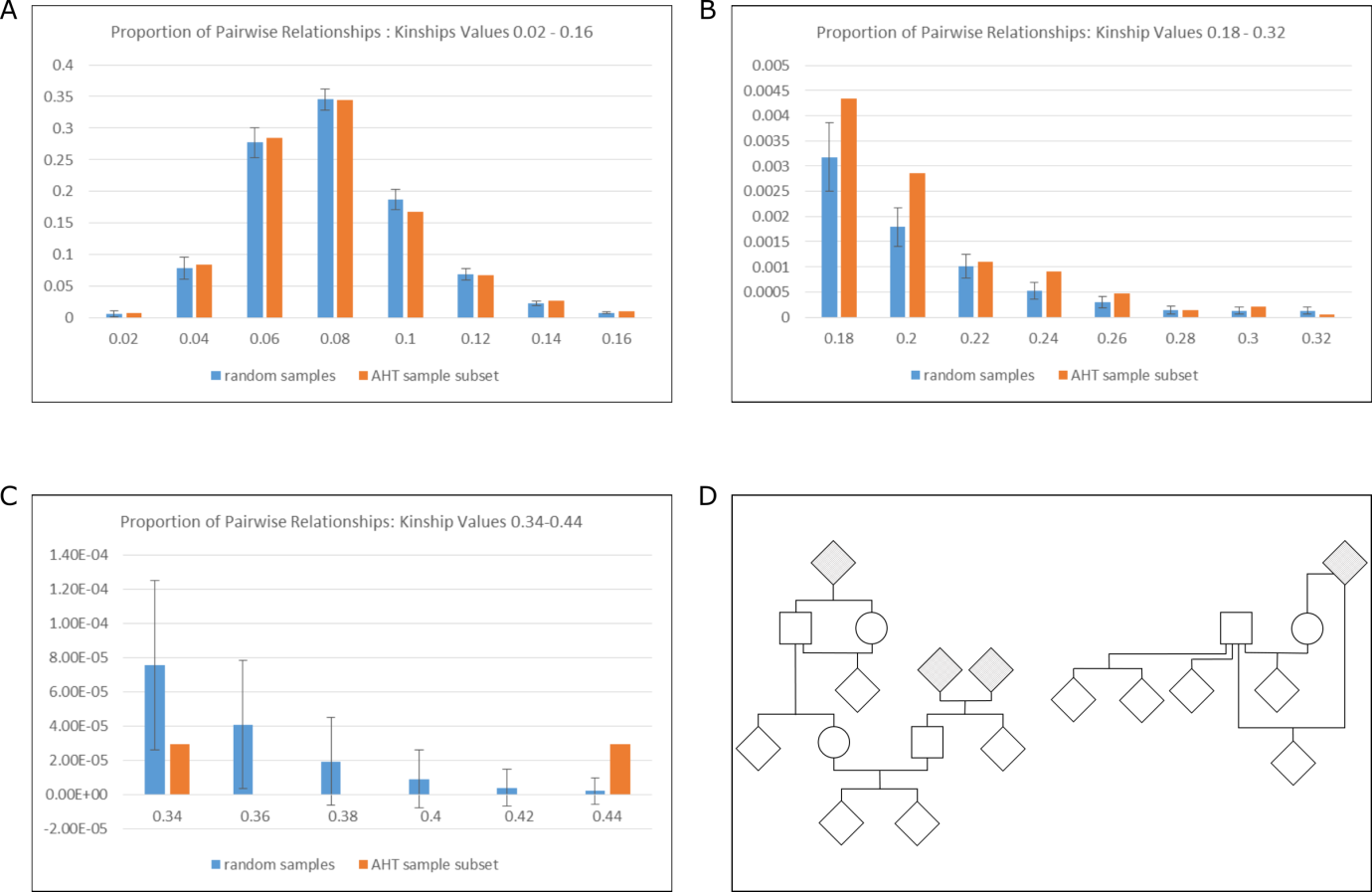


Supplementary Figure 5: (A-C) Histograms showing the proportion of pairwise relationships across the random sample sets and the AHT PRA4 DNA tested subset; (D) Pedigree drawing of the 16 outliers belonging to two distinct families: circle = female, square = male, diamond = unknown, shaded diamond = not included in our data set.


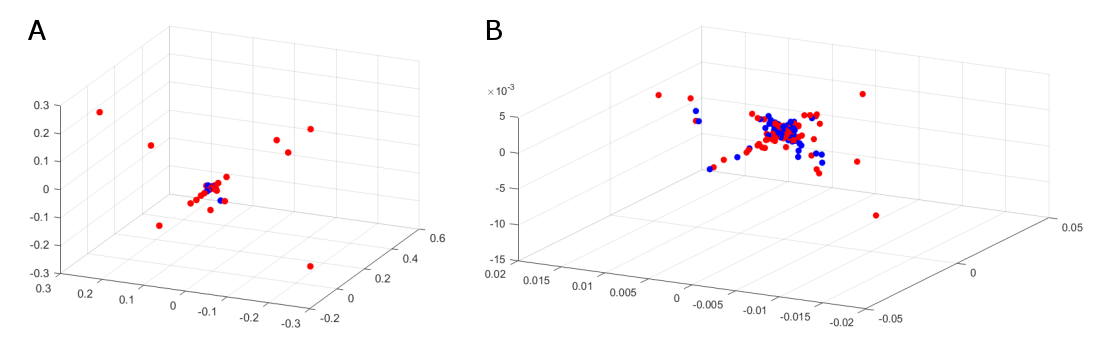


Supplementary Figure 6: (A) Multi-dimensional scaling plot to determine relatedness within each sample set. Red points represent the 261 AHT PRA4 tested samples, blue points represent 1000 randomly selected KC registered dogs born 2009-2017); (B) zoomed in on central cluster in (A) showing the main body of the AHT sample set (red) is representative of a random sample (blue).

**References**

1. Sidjanin DJ, Lowe JK, McElwee JL, Milne BS, Phippen TM, Sargan DR, Aguirre GD, Acland GM, Ostrander EA: Canine CNGB3 mutations establish cone degeneration as orthologous to the human achromatopsia locus ACHM3. *Human molecular genetics* 2002, 11(16):1823-1833.

2. Yeh CY, Goldstein O, Kukekova AV, Holley D, Knollinger AM, Huson HJ, Pearce-Kelling SE, Acland GM, Komaromy AM: Genomic deletion of CNGB3 is identical by descent in multiple canine breeds and causes achromatopsia. *BMC genetics* 2013, 14:27.

3. Tanaka N, Dutrow EV, Miyadera K, Delemotte L, MacDermaid CM, Reinstein SL, Crumley WR, Dixon CJ, Casal ML, Klein ML *et al*: Canine CNGA3 Gene Mutations Provide Novel Insights into Human Achromatopsia-Associated Channelopathies and Treatment. *PloS one* 2015, 10(9):e0138943.

4. Aguirre GD, Baldwin V, Pearce-Kelling S, Narfstrom K, Ray K, Acland GM: Congenital stationary night blindness in the dog: common mutation in the RPE65 gene indicates founder effect. *Molecular vision* 1998, 4:23.

5. Veske A, Nilsson SE, Narfstrom K, Gal A: Retinal dystrophy of Swedish briard/briard-beagle dogs is due to a 4-bp deletion in RPE65. *Genomics* 1999, 57(1):57-61.

6. Guziewicz KE, Zangerl B, Lindauer SJ, Mullins RF, Sandmeyer LS, Grahn BH, Stone EM, Acland GM, Aguirre GD: Bestrophin gene mutations cause canine multifocal retinopathy: a novel animal model for best disease. *Investigative ophthalmology & visual science* 2007, 48(5):1959-1967.

7. Zangerl B, Wickstrom K, Slavik J, Lindauer SJ, Ahonen S, Schelling C, Lohi H, Guziewicz KE, Aguirre GD: Assessment of canine BEST1 variations identifies new mutations and establishes an independent bestrophinopathy model (cmr3). *Molecular vision* 2010, 16:2791-2804.

8. Lippmann T, Jonkisz A, Dobosz T, Petrasch-Parwez E, Epplen JT, Dekomien G: Haplotype-defined linkage region for gPRA in Schapendoes dogs. *Molecular vision* 2007, 13:174-180.

9. Dekomien G, Vollrath C, Petrasch-Parwez E, Boeve MH, Akkad DA, Gerding WM, Epplen JT: Progressive retinal atrophy in Schapendoes dogs: mutation of the newly identified CCDC66 gene. *Neurogenetics* 2010, 11(2):163-174.

10. Ahonen SJ, Arumilli M, Lohi H: A CNGB1 frameshift mutation in Papillon and Phalene dogs with progressive retinal atrophy. *PloS one* 2013, 8(8):e72122.

11. Zhang Q, Acland GM, Parshall CJ, Haskell J, Ray K, Aguirre GD: Characterization of canine photoreceptor phosducin cDNA and identification of a sequence variant in dogs with photoreceptor dysplasia. *Gene* 1998, 215(2):231-239.

12. Zangerl B, Goldstein O, Philp AR, Lindauer SJ, Pearce-Kelling SE, Mullins RF, Graphodatsky AS, Ripoll D, Felix JS, Stone EM *et al*: Identical mutation in a novel retinal gene causes progressive rod-cone degeneration in dogs and retinitis pigmentosa in humans. *Genomics* 2006, 88(5):551-563.

13. Kijas JW, Cideciyan AV, Aleman TS, Pianta MJ, Pearce-Kelling SE, Miller BJ, Jacobson SG, Aguirre GD, Acland GM: Naturally occurring rhodopsin mutation in the dog causes retinal dysfunction and degeneration mimicking human dominant retinitis pigmentosa. *Proceedings of the National Academy of Sciences of the United States of America* 2002, 99(9):6328-6333.

14. Downs LM, Wallin-Hakansson B, Boursnell M, Marklund S, Hedhammar A, Truve K, Hubinette L, Lindblad-Toh K, Bergstrom T, Mellersh CS: A frameshift mutation in golden retriever dogs with progressive retinal atrophy endorses SLC4A3 as a candidate gene for human retinal degenerations. *PloS one* 2011, 6(6):e21452.

15. Downs LM, Wallin-Hakansson B, Bergstrom T, Mellersh CS: A novel mutation in TTC8 is associated with progressive retinal atrophy in the golden retriever. *Canine Genet Epidemiol* 2014, 1:4.

16. Wiik AC, Ropstad EO, Ekesten B, Karlstam L, Wade CM, Lingaas F: Progressive retinal atrophy in Shetland sheepdog is associated with a mutation in the CNGA1 gene. *Animal genetics* 2015, 46(5):515-521.

17. Clements PJ, Gregory CY, Peterson-Jones SM, Sargan DR, Bhattacharya SS: Confirmation of the rod cGMP phosphodiesterase beta subunit (PDE beta) nonsense mutation in affected rcd-1 Irish setters in the UK and development of a diagnostic test. *Current eye research* 1993, 12(9):861-866.

18. Suber ML, Pittler SJ, Qin N, Wright GC, Holcombe V, Lee RH, Craft CM, Lolley RN, Baehr W, Hurwitz RL: Irish setter dogs affected with rod/cone dysplasia contain a nonsense mutation in the rod cGMP phosphodiesterase beta-subunit gene. *Proceedings of the National Academy of Sciences of the United States of America* 1993, 90(9):3968-3972.

19. Dekomien G, Runte M, Godde R, Epplen JT: Generalized progressive retinal atrophy of Sloughi dogs is due to an 8-bp insertion in exon 21 of the PDE6B gene. *Cytogenetics and cell genetics* 2000, 90(3-4):261-267.

20. Petersen-Jones SM, Entz DD, Sargan DR: cGMP phosphodiesterase-alpha mutation causes progressive retinal atrophy in the Cardigan Welsh corgi dog. *Investigative ophthalmology & visual science* 1999, 40(8):1637-1644.

21. Mellersh CS, Boursnell ME, Pettitt L, Ryder EJ, Holmes NG, Grafham D, Forman OP, Sampson J, Barnett KC, Blanton S *et al*: Canine RPGRIP1 mutation establishes cone-rod dystrophy in miniature longhaired dachshunds as a homologue of human Leber congenital amaurosis. *Genomics* 2006, 88(3):293-301.

22. Narfstrom K, Jeong M, Hyman J, Madsen RW, Bergstrom TF: Assessment of hereditary retinal degeneration in the English springer spaniel dog and disease relationship to an RPGRIP1 mutation. *Stem cells international* 2012, 2012:685901.

23. Forman OP, Hitti RJ, Boursnell M, Miyadera K, Sargan D, Mellersh C: Canine genome assembly correction facilitates identification of a MAP9 deletion as a potential age of onset modifier for RPGRIP1-associated canine retinal degeneration. *Mammalian genome : official journal of the International Mammalian Genome Society* 2016, 27(5-6):237-245.

24. Downs LM, Bell JS, Freeman J, Hartley C, Hayward LJ, Mellersh CS: Late-onset progressive retinal atrophy in the Gordon and Irish Setter breeds is associated with a frameshift mutation in C2orf71. *Animal genetics* 2013, 44(2):169-177.

25. Downs LM, Mellersh CS: An Intronic SINE insertion in FAM161A that causes exon-skipping is associated with progressive retinal atrophy in Tibetan Spaniels and Tibetan Terriers. *PloS one* 2014, 9(4):e93990.

26. Kropatsch R, Petrasch-Parwez E, Seelow D, Schlichting A, Gerding WM, Akkad DA, Epplen JT, Dekomien G: Generalized progressive retinal atrophy in the Irish Glen of Imaal Terrier is associated with a deletion in the ADAM9 gene. *Molecular and cellular probes* 2010, 24(6):357-363.

27. Wiik AC, Wade C, Biagi T, Ropstad EO, Bjerkas E, Lindblad-Toh K, Lingaas F: A deletion in nephronophthisis 4 (NPHP4) is associated with recessive cone-rod dystrophy in standard wire-haired dachshund. *Genome research* 2008, 18(9):1415-1421.

28. Acland GM, Ray K, Mellersh CS, Langston AA, Rine J, Ostrander EA, Aguirre GD: A novel retinal degeneration locus identified by linkage and comparative mapping of canine early retinal degeneration. *Genomics* 1999, 59(2):134-142.

29. Goldstein O, Kukekova AV, Aguirre GD, Acland GM: Exonic SINE insertion in STK38L causes canine early retinal degeneration (erd). *Genomics* 2010, 96(6):362-368.
